# An Achiral Tetradentate *Cis*‐α‐Coordinating NCCN Ligand Gives Rise to a Configurationally Stable Chiral‐at‐Iron Complex for Enantioselective Catalysis

**DOI:** 10.1002/chem.202503221

**Published:** 2025-12-12

**Authors:** Lukas Hinterlang, Nemrud Demirel, Sergei I. Ivlev, Eric Meggers

**Affiliations:** ^1^ Fachbereich Chemie Philipps‐Universität Marburg Marburg Germany

**Keywords:** asymmetric catalysis, chiral iron complex, chiral‐at‐metal, mesoionic carbenes, tetradentate ligand

## Abstract

Ligand design plays a crucial role in developing chiral transition metal complexes with enhanced or new catalytic properties. Here, we report our progress toward a new class of linear tetradentate NCCN ligands incorporating strongly σ‐donating carbene moieties. The NCCN ligand coordinates to iron(II) in a *cis*‐α topology, with two pyridine donors occupying the apical positions and two 1,2,3‐triazolin‐5‐ylidene mesoionic carbene (MIC) donors in the equatorial plane. Two acetonitrile ligands complete the octahedral coordination sphere, and their lability provides the basis for the observed catalytic activity. Notably, the two strongly σ‐donating MIC groups create a strong ligand field, which is critical for achieving configurational stability of the metal‐centered stereogenicity. This design strategy thus enabled the first chiral‐at‐iron catalyst derived from an achiral tetradentate ligand, which was applied to catalytic and enantioselective C(sp^3^)−H amination and a Cannizzaro reaction.

## Graphical abstract

The design and synthesis of a linear tetradentate NCCN ligand incorporating two 1,2,3‐triazolin‐5‐ylidene mesoionic carbene donors, and its application to asymmetric iron catalysis is reported. This work represents the first example of an iron complex derived from a *cis*‐α‐coordinating tetradentate NCCN ligand, and notably, the first chiral iron catalyst derived from an achiral tetradentate ligand.

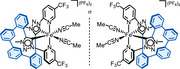

## 1 Introduction

Chiral transition metal complexes represent a highly versatile class of catalysts for asymmetric transformations [1]. This versatility arises from the vast diversity of available metals in various oxidation states, different coordination geometries, and the wide range of possible ligand frameworks [2]. Together, these factors enable numerous activation modes, catalytic mechanisms, and strategies for asymmetric induction. Consequently, ligand design plays a pivotal role in fine‐tuning the properties and reactivity of chiral transition metal catalysts.

**FIGURE 1:**
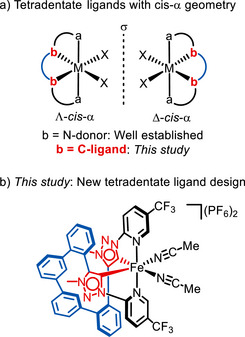
Background and this study. (a) Mirror‐imaged metal complexes with tetradentate ligands in *cis*‐α configuration. (b) This study: Chiral‐at‐iron catalyst with tetradentate ligand containing two mesoionic carbene ligands.

Among important ligand scaffolds, linear tetradentate ligands complemented by two ancillary monodentate ligands have gained considerable popularity for generating chiral octahedral transition metal catalysts [3]. The tetradentate ligands are often symmetric, which offers the advantage that *cis*‐α coordination results in *C*_2_ symmetry (Figure 1a). This *C*_2_ symmetry is usually beneficial for asymmetric catalysis, as it reduces the number of competing transition states during the catalytic cycle. In addition, *cis*‐α coordination gives rise to an overall helical topology with metal‐centered chirality, which governs asymmetric induction during catalysis [4]. The tetradentate *cis*‐α coordination framework is especially attractive for catalysts based on earth‐abundant metals [5, 6, 7, 8], where the chelate effect compensates for the typically more labile coordinative metal–ligand bonds and thus secures the composition of the complexes during catalysis. In the case of catalysts from iron [9, 10, 11, 12, 13, 14, 15, 16, 17, 18, 19, 20], which are of current high interest due to economical and environmental aspects, the most widely used linear tetradentate ligands are of the type bis(iminomethyl)diamine (N4 ligands), where the imine donors (pyridine, quinoline, benzimidazole, etc.) occupy the apical positions and the tertiary amines occupy the equatorial positions [7, 8, 9, 10, 11, 12, 13, 14, 15, 16, 17, 18, 19, 20]. These ligands are almost invariably chiral, allowing the chiral backbone to dictate the configuration of the metal center formed upon coordination [3].

N‐Heterocyclic carbenes (NHCs) are among the most versatile ligands, as their strong σ‐donating abilities promote the formation of robust metal‐ligand bonds and increase the electron density at the metal center, thereby modulating reactivity and catalytic performance [21]. Consequently, considerable effort has been devoted to the development of multidentate NHC ligands. With respect to iron [22, 23, 24, 25, 26], Danopoulos first introduced iron pincer complexes based on the ligand 2,6‐bis(imidazolylidene)pyridine [27]. Subsequently, Hahn and co‐workers reported iron complexes featuring N‐pyridyl‐substituted imidazolin‐2‐ylidenes (PyNHC) [28]. Building on this work, Chen and co‐workers linked two PyNHC units via a methylene bridge to generate a tetradentate NCCN ligand, leading to the formation of a square‐planar 14‐valence‐electron iron complex [29]. Kühn and co‐workers expanded this concept by varying the length of the alkylene spacer in NCCN ligands [30]. Methylene and ethylene linkers produced square‐planar coordination of the NCCN ligand to iron(II), with two *trans*‐coordinated acetonitriles completing the octahedral coordination sphere. In contrast, a propylene bridge yielded a sawhorse‐type *cis*‐β coordination geometry with two *cis*‐coordinated acetonitriles. Inspired by the *cis*‐α coordination geometry of the aforementioned nonheme iron(II) catalysts, Glorius and co‐workers reported a tetradentate CNNC ligand incorporating two imidazo[1,5‐*a*]pyridine‐3‐ylidene units connected via a diamine linker [31]. This ligand coordinates to iron in a highly distorted octahedral geometry characterized by weak Fe−N interactions. A *C*_2_‐symmetric *cis*‐α‐coordinating NCCN ligand has been elusive.

Herein, we report a design strategy to provide multidentate ligands incorporating carbene donor sites. Specifically, we describe the design and synthesis of a linear tetradentate NCCN ligand featuring two 1,2,3‐triazolin‐5‐ylidene mesoionic carbene (MIC) donors, along with its application in asymmetric iron catalysis (Figure 1b). Remarkably, despite the absence of chirality in the ligand framework, a *C*_2_‐symmetric, configurationally inert chiral iron catalyst is generated through *cis*‐α coordination. To the best of our knowledge, this work represents the first example of an iron complex derived from a *cis*‐α‐coordinating tetradentate NCCN ligand, and notably, the first chiral iron catalyst derived from an achiral tetradentate ligand (chiral‐at‐iron catalyst).

## 2 Results and Discussion

### 2.1 Design Principle

**FIGURE 2:**
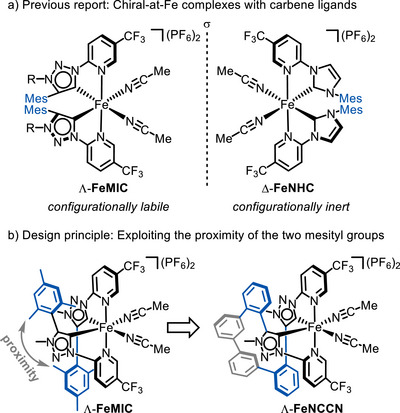
Design rationale. (a) Previous chiral‐at‐iron catalysts containing two pyridyl‐substituted carbene ligands. (b) Linker design by replacing two methyl groups of the adjacent mesityl groups with a biphenyl linker (shown in gray) to construct a new tetradentate ligand.

We recently introduced a novel class of chiral iron catalysts which contain exclusively achiral ligands, with overall chirality arising from a stereogenic iron center (chiral‐at‐iron catalysts) [32, 33, 34]. In our initial design, the iron(II) center is *cis*‐coordinated by two N‐(2‐pyridyl)‐substituted N‐heterocyclic carbene (PyNHC) ligands in a bidentate fashion, along with two monodentate acetonitrile ligands. The resulting dicationic complex is paired with two hexafluorophosphate counterions (Figure 2a, **FeNHC**). Depending on the helical twist of the PyNHC ligands, the metal center adopts either a Λ‐ or Δ‐absolute configuration. Notably, the two PyNHC ligands are both constitutionally and configurationally inert, while the coordinated acetonitriles are labile, enabling asymmetric transition metal catalysis without the need to introduce chirality into the ligand sphere. However, replacing the NHC moieties with 1,2,3‐triazolin‐5‐ylidene mesoionic carbene (MIC) ligands renders the iron center configurationally labile (**FeMIC**) [35, 36]. Given that MIC ligands are strong σ‐donors and impart distinct catalytic properties, we sought strategies to lock the metal‐centered stereogenicity [37, 38, 39, 40, 41, 42, 43, 44, 45]. Previously, we reported our efforts to achieve this using chiral pyridyl MIC ligands [35, 36]. In the present work, we pursued an alternative approach by converting the bidentate pyridyl MIC ligands into a tetradentate framework.

In the structure of **FeMIC**, the two mesityl groups engage in interligand π‐stacking, bringing two methyl groups of the mesityl moieties into close proximity (Figure 2b, left). Simple molecular modeling suggested that replacing these two methyl groups with a biphenyl moiety could provide an effective linker, thus providing a new class of tetradentate ligands (Figure 2b, right). For the sake of simplicity, we also removed the remaining methyl substituents on the mesityl groups, resulting in two MIC moieties connected by a chain of four benzene moieties.

### 2.2 Ligand Synthesis and Iron Coordination

**SCHEME 1:**
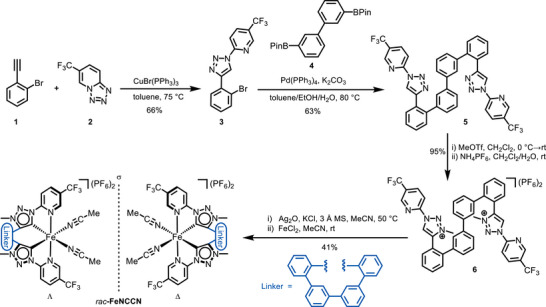
Ligand synthesis and iron coordination to generate *rac*‐**FeNCCN**. Pin = pinacolato, OTf = trifluoromethanesulfonate, MS = molecular sieves.

The ligand synthesis commenced from commercially available 1‐bromo‐2‐ethynylbenzene **1**, which underwent a copper‐catalyzed azide‐alkyne cycloaddition with tetrazole **2** to afford triazole **3** in 66% yield (Scheme 1) [46]. This triazole intermediate was subsequently subjected to a double Suzuki cross‐coupling with biphenyldiborane **4**, providing coupling product **5** in 69% yield [47]. In the final step, double methylation of **5** furnished the bis‐triazolium salt **6** as its hexafluorophosphate salt in 95% yield [36].

The bis‐triazolium salt **5** was then employed in the synthesis of a corresponding iron(II) complex. For this purpose, an established Ag‐carbene route was chosen, previously utilized by our group for related iron MIC complexes [35, 36]. The triazolium salt was first converted into a silver‐carbene complex using silver oxide in the presence of KCl, followed by transmetalation with FeCl_2_ as the iron source. A reaction time of 3 h and reduced concentration were found to be optimal for the coordination step, likely minimizing oligomerization. Under these conditions, the racemic complex *rac*‐**FeNCCN** was isolated in 41% yield (Scheme 1). The racemic complex *rac*‐**FeNCCN** is air‐stable and shows no signs of decomposition when stored in acetonitrile under ambient conditions over the time period of three weeks (see Supporting Information for details). However, when the complex was stored in dichloromethane, signs of decomposition appeared after two days, indicating a somewhat reduced stability in this noncoordinating solvent.

### 2.3 Crystal Structure

**FIGURE 3:**
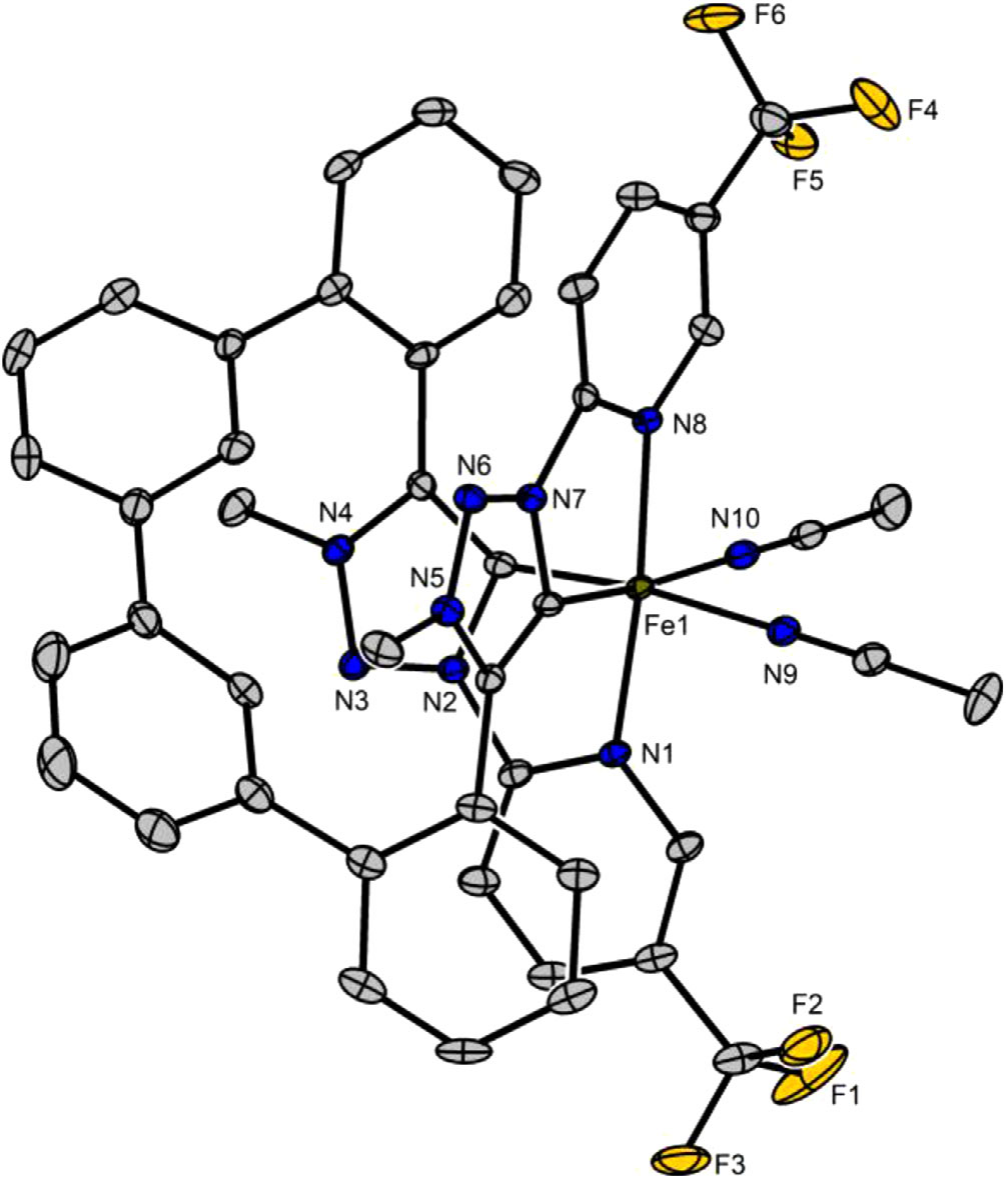
Crystal structure of the racemic iron complex *rac*‐**FeNCCN**. Counter ions and the hydrogen atoms are not shown. Displacement ellipsoids are shown at a 50% probability level at 100 K.

To confirm the molecular structure, single crystals suitable for X‐ray diffraction were obtained. The analysis revealed the expected tetradentate coordination of the NCCN ligand with two equatorially coordinated acetonitrile ligands completing the octahedral coordination sphere (Figure 3). Two hexafluorophosphate anions balance the charge of the dicationic iron complex. The pyridyl donors of the tetradentate ligand occupy the axial positions, while the two MIC ligands are located in the equatorial plane, giving rise to a *C*_2_‐symmetric *cis*‐α topology. The helical twist of the tetradentate ligand generates metal‐centered chirality. Notably, the Ph_4_‐linker is well accommodated within the coordination environment, although slight distortion of the octahedral coordination geometry suggests that the linker is slightly too large.

### 2.4 Nonracemic Complexes

**FIGURE 4:**
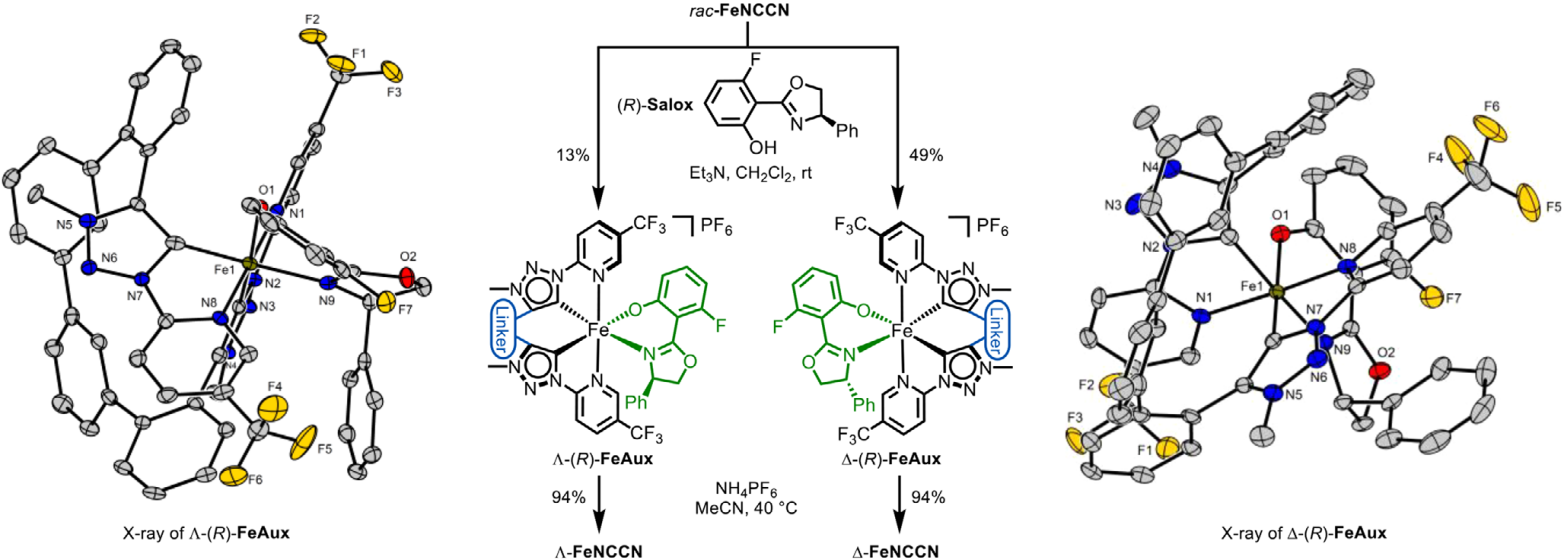
Chiral‐auxiliary‐mediated synthesis of the nonracemic iron complexes Λ‐ and Δ‐**FeNCCN**. Crystal structures of Λ‐ and Δ‐(*R*)‐**FeAux**: Counter ions and the hydrogen atoms are not shown. Displacement ellipsoids are shown at 50% probability level at 100 K.

**FIGURE 5:**
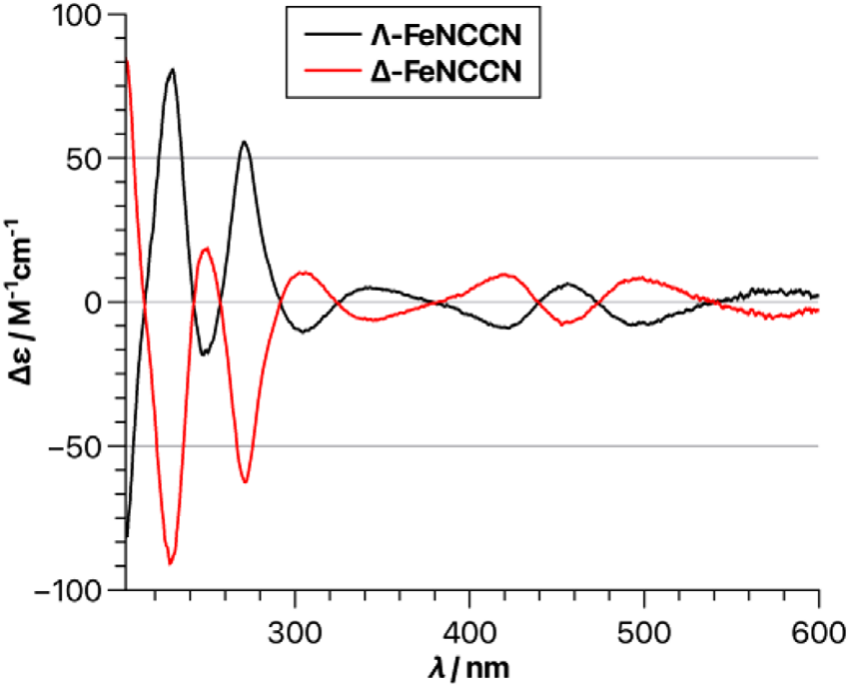
CD‐spectra of Λ‐**FeNCCN** and Δ‐**FeNCCN** in MeCN (0.25 mM).

The racemic mixture of *rac*‐**FeNCCN** was resolved into its individual enantiomers using a chiral‐auxiliary approach [4, 48, 49]. Specifically, the chiral salicyloxazoline derivative (*R*)‐**Salox** [50] was reacted with *rac*‐**FeNCCN** under basic conditions to afford a mixture of the diastereomeric complexes Λ‐(*R*)‐**FeAux** and Δ‐(*R*)‐**FeAux** (Figure 4). ^19^F NMR spectroscopy revealed a 1:1 ratio of both diastereomers in solution. However, following column chromatographic separation, Δ‐(*R*)‐**FeAux** was isolated in 49% yield, while Λ‐(*R*)‐**FeAux** was obtained in only 13% yield. The lower yield of the Λ‐diastereomer, which also contains small amounts of impurities, is attributed to its reduced stability and partial decomposition on silica gel (see Supporting Information for details). Single‐crystal X‐ray diffraction analysis of both auxiliary complexes confirmed the opposite metal‐centered configurations. Importantly, in Δ‐(*R*)‐**FeAux**, the phenyl substituent of the oxazoline moiety engages in favorable π–π stacking with one of the pyridyl ligands, an interaction absent in Λ‐(*R*)‐**FeAux**, thereby rationalizing the difference in stability of the two diastereomers. Finally, cleavage of the auxiliary ligands under acidic conditions provided the individual enantiomers Λ‐**FeNCCN** and Δ‐**FeNCCN** (94% each), which displayed mirror‐image circular dichroism (CD) spectra (Figure 5). Taken together, Δ‐**FeNCCN** is accessible conveniently via the chiral auxiliary (*R*)‐**Salox**. However, because Λ‐(*R*)‐**FeAux** cannot be obtained in high yield and is difficult to isolate in pure form, it is preferable to prepare Λ‐**FeNCCN** from the enantiomeric auxiliary (*S*)‐**Salox**.

**FIGURE 6:**
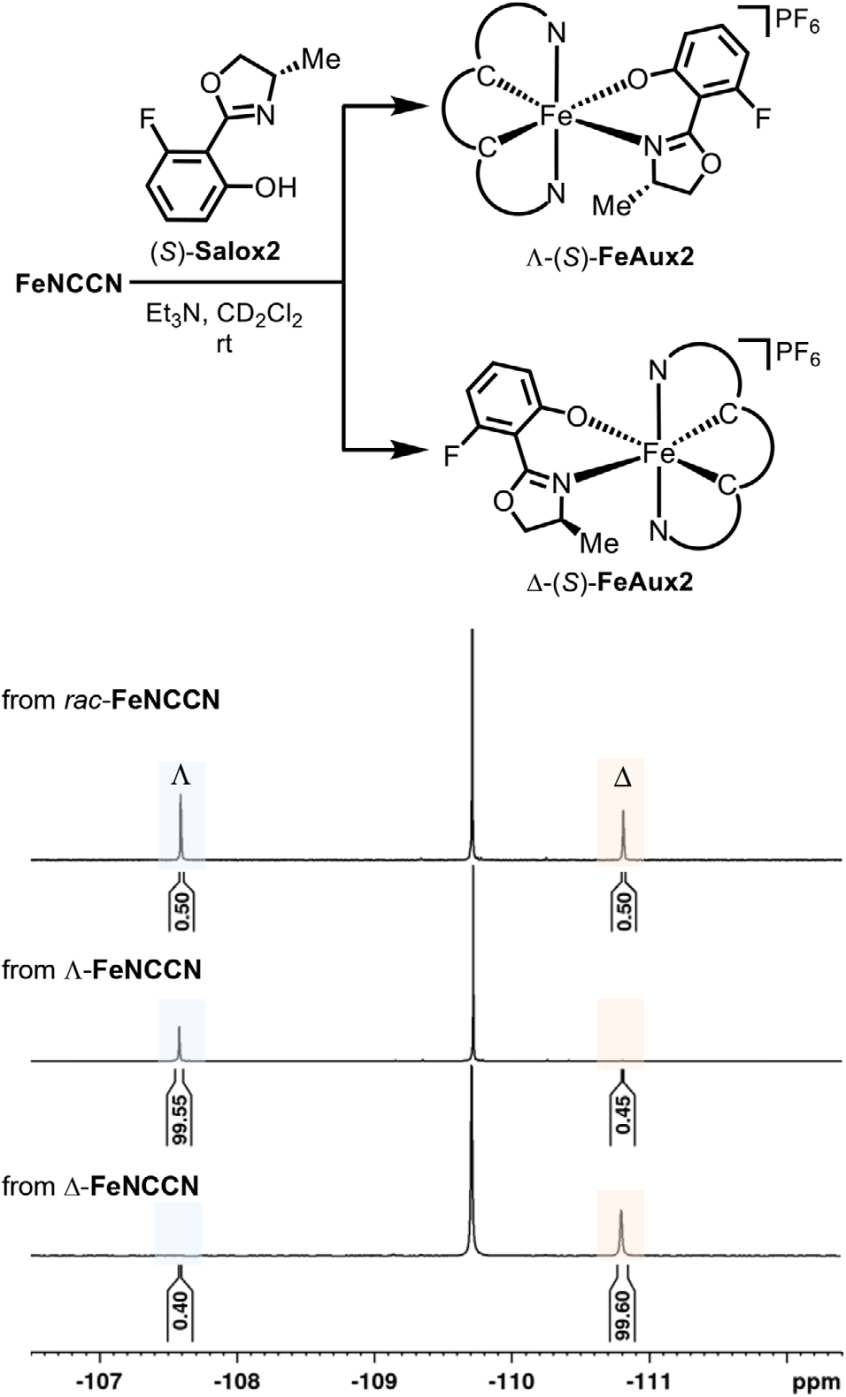
Determination of the enantiomeric purity of Λ‐ and Δ‐**FeNCCN**. The complexes were reacted with the chiral ligand (*S*)‐**Salox2,** and the resulting complexes were analyzed by ^19^F NMR spectroscopy.

The enantiomeric purity of the complexes Λ‐**FeNCCN** and Δ‐**FeNCCN** was verified by ^19^F NMR spectroscopy following coordination with an excess of the chiral ligand (*S*)‐**Salox2** under basic conditions. This reaction converts the two enantiomers of **FeNCCN** into the corresponding diastereomers Λ‐(*S*)‐**FeAux2** and Δ‐(*S*)‐**FeAux2**, which can be readily distinguished by ^19^F NMR spectroscopy (Figure 6) [51]. Owing to the small sample quantities required, the straightforward in‐tube procedure, and the high sensitivity of ^19^F NMR spectroscopy, this represents an efficient and reliable method for assessing the enantiomeric purity of such complexes [52]. As a reference, first the racemic complex was treated with the chiral auxiliary, yielding a 1:1 mixture of the diastereomers Λ‐(*S*)‐**FeAux2** and Δ‐(*S*)‐**FeAux2** (Figure 6a), thereby confirming complete conversion of both enantiomers. In contrast, reactions starting from either enantiopure Λ‐ or Δ‐**FeNCCN** afforded diastereomeric ratios exceeding 99:1 (Figure 6b), thus establishing the configurational stability of **FeNCCN**. These findings underscore the role of the Ph_4_‐linker in stabilizing the iron MIC complex and preventing racemization, which could not be achieved with the original complex **FeMIC** containing only bidentate and monodentate ligand [35].

### 2.5 Asymmetric Catalysis

With enantiomerically pure chiral‐at‐iron **FeNCCN** in hand, we next explored its catalytic properties. **FeNCCN** provides access to a chiral‐at‐iron triazolinylidene complex as a single enantiomer, which was not achievable with its predecessor **FeMIC** [35]. In our recent studies, we demonstrated that the more electron‐rich iron MIC complex exhibits higher reactivity in a ring‐closing C(sp^3^)−H amidation compared to the corresponding imidazolylidene congeners [35]. Accordingly, using Δ‐**FeNCCN** as the catalyst in this reaction, we achieved a high conversion of 90% of the urea derivative **7**, although the ^1^H NMR yield of the desired ring‐closing C(sp^3^)−H amidation product 2‐imidazolidinone **8** was only modest with 32% yield, with a moderate enantiomeric excess of 59% ee (Table 1, entry 1) [53, 54, 55]. Similar low yields were observed for the bis‐bidentate complex **FeMIC** (R = Me) [35]. The main side product in this reaction is an acyclic urea devoid of the benzoate group. At a lower temperature, this side reaction is suppressed. Accordingly, a reduced temperature of 4°C led to a lower conversion of 60%, although the yield could be increased to 45% without any significant change in the enantiomeric excess (entry 2). An even lower temperature of −15°C improved the yield to 54% (64% conversion) but with only a slight improvement of the enantioselectivity (61% ee) (entry 3).

**TABLE 1:** C(*sp* ^3^)‐H‐amidation of benzoyloxyurea **7** with Δ‐**FeNCCN** to 2‐imidazolidinone **8**.^[^ ^a^ ^]^

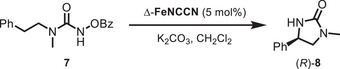					
Entry	Time [h]	T [°C]	Conv. [%]	Yield^[^ ^b^ ^]^	ee^[^ ^c^ ^]^
1	24	rt	90	32%	59%
2	24	4	60	45%	59%
3	24	−15	64	54%	61%

^[a]^ Reaction conditions: Substrate **7** (0.03 mmol), Δ‐**FeNCCN** (5 mol%), and K_2_CO_3_ (0.10 mmol) were placed in CH_2_Cl_2_ (0.05 M) under N_2_. Then, stirring was carried out for the indicated time and at the indicated temperature.

^[b]^ Determined by ^1^H‐NMR analysis of the crude product with TMB as internal standard.

^[c]^ Enantiomeric excess of the crude product determined by HPLC analysis on a chiral stationary phase.

**SCHEME 2:**
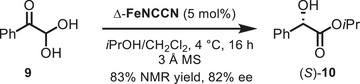
Application of Δ‐**FeNCCN** to an enantioselective Cannizzaro reaction.

Next, we applied Δ‐**FeNCCN** to the intramolecular Cannizzaro [56, 57] reaction of phenylglyoxal **9** to the mandelate ester **10**, verifying its potential in Lewis‐acid catalysis (Scheme 2). Accordingly, at 4°C, Δ‐**FeNCCN** exhibited high activity, affording an NMR yield of 83% and an enantiomeric excess of 82% ee.

## 3 Conclusions

Ligand design plays a pivotal role in the development of chiral transition metal complexes with enhanced or even unprecedented catalytic properties. Motivated by this, we here report our progress in developing a new class of linear tetradentate NCCN ligands featuring strongly σ‐donating carbene moieties, distinguishing them from the well‐studied N4 ligand frameworks. In this study, the developed NCCN ligand coordinates to iron(II) in a *cis*‐α topology, with two pyridines occupying the axial positions and two 1,2,3‐triazolin‐5‐ylidene mesoionic carbene (MIC) donors in the equatorial plane. Two acetonitrile molecules complete the octahedral coordination sphere, and their lability provides the basis for catalytic activity. Notably, the two strongly σ‐donating MIC groups generate a strong ligand field, which is essential for achieving configurational inertness and establishing metal‐centered chirality. This design therefore, enabled the first chiral‐at‐iron catalyst derived from an achiral tetradentate ligand. Of note, while Sauvage and Lacour introduced, over twenty years ago, a chiral‐at‐iron complex based on an achiral tetradentate bis‐2,9‐phenanthroline framework [58], the absence of labile ligands in their complex prevents applications as chiral catalysts. Ongoing work in our laboratory focuses on further developing this novel class of NCCN ligands for applications in asymmetric catalysis.

## Conflicts of Interest

The authors declare not competing financial interest.

## Supporting information




**Supporting file 1**: The authors have cited additional references within the Supporting Information [59, 60, 61, 62, 63, 64]. The Supporting Information includes detailed experimental procedures, analytical data, NMR spectra, CD spectra, and HPLC traces. Deposition numbers 2497296 (for *rac*‐**FeNCCN**), 2497297 (for Λ‐(*R*)‐**FeAux**), and 2497298 (for Δ‐(*R*)‐**FeAux**) contain the supplementary crystallographic data for this paper. These data are provided free of charge by the joint Cambridge Crystallographic Data Centre and Fachinformationszentrum Karlsruhe Access Structures service.

## Data Availability

The data supporting this article are included as part of the Supplementary Information.
