# Supplementary material for: An Achiral Tetradentate Cis‐α‐Coordinating NCCN Ligand Gives Rise to a Configurationally Stable Chiral‐at‐Iron Complex for Enantioselective Catalysis
Source: Chemistry. 2025 Dec 12;32(3):e03221. doi: 10.1002/chem.202503221 (PMC12824824; doi:10.1002/chem.202503221)
Supplement: Supplementary file 1 — Supporting file 1: The authors have cited additional references within the Supporting Information [59, 60, 61, 62, 63, 64]. The Supporting Information includes detailed experimental procedures, analytical data, NMR spectra, CD spectra, and HPLC traces. Deposition numbers 2497296 (for rac‐FeNCCN), 2497297 (for Λ‐(R)‐FeAux), and 2497298 (for Δ‐(R)‐FeAux) contain the supplementary crystallographic data for this paper. These data are provided free of charge by the joint Cambridge Crystallographic Data Centre and Fachinformationszentrum Karlsruhe Access Structures service. [file CHEM-32-e03221-s001.pdf]

Supporting Information

An Achiral Tetradentate *cis*-α-Coordinating NCCN Ligand Gives Rise to a Configurationally Stable Chiral-at-Iron Complex for Enantioselective Catalysis

Lukas Hinterlang, Nemrud Demirel, Sergei I. Ivlev, and Eric Meggers*

Fachbereich Chemie, Philipps-Universität Marburg, Hans-Meerwein-Strasse 4, 35043 Marburg, Germany

*Email: meggers@chemie.uni-marburg.de

Table of Contents

1. General Information 1

2. Ligand Synthesis 2

3. Synthesis of Racemic Iron Complex 5

4. Synthesis of Auxiliary Complexes 6

5. Cleavage of the Chiral Auxiliary 8

6. Determination of Enantiomeric Excess of Λ- and Δ-FeNCCN 10

7. Stability Experiments 11

8. Catalysis 12

9. NMR Spectra 14

10. Chiral HPLC Traces 23

11. CD-Spectra 24

12. Single Crystal X-Ray Diffraction 25

13. References 31

1. General Information

All reactions were carried out under a nitrogen atmosphere in oven-dried glassware unless noted otherwise. Dry solvents for sensitive reactions were dried according to standard purification methods using calcium hydride (MeCN, CH_2Cl_2, MeOH), phosphorus pentoxide ($CHCl_3$), sodium (THF, Et_2O, toluene) and distilled under an atmosphere of nitrogen. The chemicals used are all from commercial sources and were used without further purification unless stated otherwise. For purification by column chromatography, Macherey-Nagel silica gel 60M (irregularly shaped, 230–400 mesh, pH 6.8, pore volume: 0.81 mL/g, mean pore size: 66 Å, specific surface: 492 m^2/g, particle size distribution: 0.5% < 25 µm and 1.7% > 71 µm, water content: 1.6%) was used as the stationary phase. ^{1}H-NMR, ^{13}C{^{1}H} NMR and ^{19}F{^{1}H} NMR spectra were recorded on a Bruker AV III HD 300 MHz, AV II 300 MHz, AV III HD 500 MHz, AV III 500 MHz, NEO 300 MHz, or NEO 600 MHz spectrometer at ambient temperature. The chemical shift δ is reported in parts per million (ppm) with the residual proton signal of the deuterated solvent as reference. ^{19}F{^{1}H} NMR spectra were calibrated to trichlorofluoromethane ($CFCl_3$, $\delta = 0$ ppm) as external standard. All infrared spectroscopy was performed on a Bruker Alpha FT−IR spectrometer. Chiral HPLC was performed on an AGILENT 1200 or AGILENT 1260 HPLC system with DAICEL columns as chiral stationary phase with a size of 4.6 x 250 mm and a particle size of 5 µm. CD-spectra were recorded on a Jasco J-810 CD spectropolarimeter from 700–200 nm, 1 nm bandwidth, 2 s response time, 50 nm/min scanning speed, and an accumulation of 3 scans. High-resolution mass spectrometry was performed on a Finnigan LTQ-FT Ultra mass spectrometer (Thermo Fischer Scientific) using Electrospray ionization (ESI) as ionization source. Tetrazole **2**[1], the chiral auxiliaries (*R*)-**Salox**[2], (*S*)-**Salox2**[2] and the substrate **7**[3] were synthesized after a modified literature procedure.

2. Ligand Synthesis

2-(4-(2-bromophenyl)-1*H*-1,2,3-triazol-1-yl)-5-(trifluoromethyl)pyridine (3)

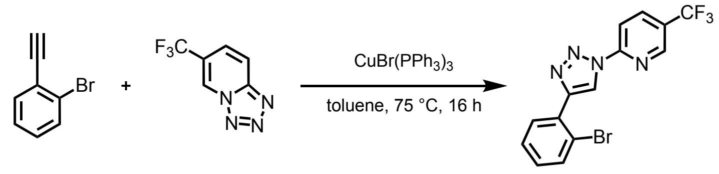

1 **2** **3**

Following a modified procedure from the literature.[4] tetrazole **2** (1.50 g, 7.97 mmol, 1.00 eq) was mixed with $CuBr(PPh_3)_3$ (0.37 g, 0.40 mmol, 0.05 eq) and dissolved in toluene (0.14 M based on the tetrazole) under an atmosphere of nitrogen, whereupon 1-bromo-2-ethynylbenzene (**1**, 1.05 mL, 8.77 mmol, 1.10 eq) was added dropwise. The reaction vessel was sealed and heated to 75 °C for 16 h. After cooling to room temperature, the mixture was filtered over celite and washed with EtOAc. The solvent was then removed under reduced pressure and the crude product was purified by flash column chromatography (silica gel, *n*-hexane/EtOAc, 99:1 → 90:10) to obtain the triazole **3** (1.93 g, 5.23 mmol, 66%) as an off-white solid.

TLC: R_f = 0.30 (*n*-hexane/EtOAc 15:1).

^{1}H-NMR: (500 MHz, $CDCl_3$) δ (ppm) = 9.27 (s, 1H), 8.82 (dt, *J* = 2.6, 0.9 Hz, 1H), 8.42 (dt, *J* = 8.6, 0.8 Hz, 1H), 8.22–8.15 (m, 2H), 7.71 (dd, *J* = 8.1, 1.2 Hz, 1H), 7.46 (td, *J* = 7.6, 1.3 Hz, 1H), 7.26 (ddd, *J* = 8.0, 7.4, 1.7 Hz, 1H).

^{13}C-NMR: (126 MHz, $CDCl_3$) δ (ppm) = 151.7, 146.6, 146.6, 137.0, 134.2, 131.1, 130.9, 128.2, 127.0, 126.7, 124.5, 121.9, 120.8, 114.1.

^{19}F-NMR: (282 MHz, $CDCl_3$) δ (ppm) = –62.20 (s, 3F).

HRMS: ESI(+); *m/z* calculated for $C_{14}H_8F_3N_4BrNa$ $[M+Na]^+$: 390.98, found: 390.9758 $[M+Na]^+$.

IR: ṽ (cm^{-1}) = 3181 (w), 3117 (w), 3086 (w), 1606 (m), 1592 (w), 1549 (w), 1493 (m), 1466 (w), 1446 (m), 1422 (w), 1382 (w), 1323 (m), 1260 (w), 1234 (w), 1220 (w), 1193 (w), 1166 (w), 1128 (s), 1102 (w), 1078 (m), 1035 (s), 1015 (w), 998 (w), 964 (w), 946 (w), 936 (w), 854 (m), 813 (m), 796 (w), 753 (s), 723 (m), 703 (m), 643 (w), 635 (w), 612 (w), 580 (w), 538 (w), 508 (w), 489 (m), 450 (w), 433 (m).

Biphenyl-linked triazole 5

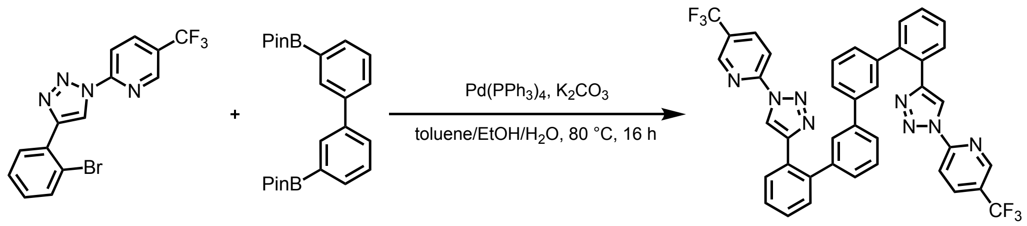

3 4 5

Following a modified procedure from the literature.[5] The arylboronic ester **4** (0.50 g, 1.23 mmol, 1.00 eq), K_2CO_3 (0.68 g, 4.92 mmol, 4.00 eq), $Pd(PPh_3)_4$ (71.1 mg, 0.06 mmol, 0.05 eq), and the triazole **3** (0.95 g, 2.58 mmol, 2.10 eq) were placed in a Schlenk tube under nitrogen atmosphere. Toluene/EtOH/H_2O (3:1:1, 0.05 M based on the boronic ester) was added and the reaction mixture was stirred at 80 °C for 16 h. After cooling to room temperature, the mixture was diluted with H_2O and extracted three times with CH_2Cl_2. The combined organic phases were then washed with brine and dried over $MgSO_4$. After filtering over a short silica pad and rinsing with EtOAc, the solution was concentrated under reduced pressure and transferred to a centrifuge tube, where the remaining solvent was removed under reduced pressure. The solid residue was washed six times with EtOAc by centrifugation, removing the supernatant each time. The resulting solid was dried under vacuo to yield the pure biphenyl-linked triazole **5** (0.62 g, 0.85 mmol, 69%) as a colorless solid.

TLC: R_f = 0.24 (CH_2Cl_2/MeOH 25:1).

^{1}H-NMR: (600 MHz, $CDCl_3$) δ (ppm) = 8.47 (dd, J = 2.2, 1.1 Hz, 1H), 8.17 (d, J = 8.6 Hz, 1H), 8.13 (dd, J = 7.7, 1.4 Hz, 1H), 8.01 (dd, J = 8.5, 2.4 Hz, 1H), 7.69 (s, 1H), 7.56 (ddd, J = 7.7, 1.9, 1.1 Hz, 1H), 7.50 (td, J = 7.5, 1.4 Hz, 1H), 7.48–7.41 (m, 3H), 7.38 (dd, J = 7.6, 1.4 Hz, 1H), 7.29–7.24 (m, 1H).

^{13}C-NMR: (151 MHz, $CDCl_3$) δ (ppm) = 151.1, 147.1, 146.1, 141.8, 141.5, 140.5, 136.5, 130.6, 129.2, 129.1, 128.8, 128.7, 128.4, 128.1, 126.6, 126.1, 124.0, 122.2, 119.5, 113.5.

^{19}F-NMR: (282 MHz, $CDCl_3$) δ (ppm) = –62.25 (s, 3F).

HRMS: ESI(+); *m/z* calculated for $C_{40}H_{24}F_6N_8H$ $[M+H]^+$: 731.20, found: 731.2109 $[M+H]^+$.

IR: ṽ (cm^{-1}) = 3173 (w), 1603 (m), 1547 (w), 1490 (m), 1469 (w), 1445 (w), 1427 (m), 1385 (w), 1325 (s), 1296 (w), 1254 (w), 1232 (w), 1218 (m), 1189 (w), 1173 (w), 1155 (w), 1133 (s), 1098 (w), 1088 (w), 1078 (w), 1034 (s), 1016 (w), 999 (m), 964 (w), 940 (w), 895 (w), 888 (w), 845 (m), 815 (w), 792 (m), 771 (s), 759 (w), 713 (m), 674 (m), 636 (w), 621 (w), 608 (w), 588 (w), 551 (w), 527 (w), 497 (w), 487 (m), 431 (w).

Bis-triazolium salt 6

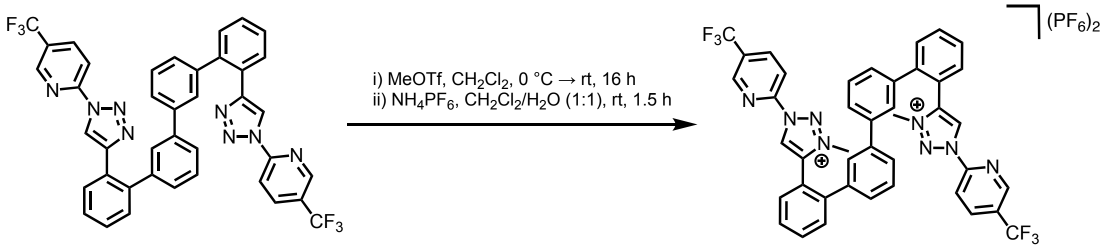

5 **6**

Following a modified procedure from the literature.[6] The biphenyl-linked triazole **5** (0.26 g, 0.36 mmol, 1.00 eq) was placed in a Schlenk tube under nitrogen atmosphere and suspended in dry CH_2Cl_2 (0.17 M based on the triazole). The mixture was cooled to 0 °C, whereupon MeOTf (98.2 µL, 0.86 mmol, 2.40 eq) was added. After stirring for 10 minutes at 0 °C, the suspension was allowed to warm to room temperature and stirred for additional 16 h. The yellowish, clear solution was then diluted with CH_2Cl_2 and filtered over a silica pad, washing out any impurities with CH_2Cl_2. The triflate salt was then eluted with CH_2Cl_2/MeOH (5:1) and, after removal of the solvent under reduced pressure, was taken up in a mixture of CH_2Cl_2/H_2O (1:1, 0.10 M based on the triazole). After adding NH_4PF_6 (0.58 g, 3.61 mmol, 10.00 eq), the mixture was stirred vigorously for 1.5 hours under air at room temperature. The organic phase was then separated, and the aqueous phase was extracted once with CH_2Cl_2. The combined organic phases were then washed with H_2O, dried over $MgSO_4$, filtered, and the solvent was removed under reduced pressure to yield the analytically pure biphenyl-linked ligand **6** (0.36 g, 0.34 mmol, 94%) as a light yellow solid.

^{1}H-NMR: (500 MHz, CD_3CN) δ (ppm) = 9.25 (s, 1H), 8.96 (dt, *J* = 2.5, 0.9 Hz, 1H), 8.53–8.47 (m, 1H), 8.26–8.20 (m, 1H), 7.83 (ddd, *J* = 7.8, 6.5, 2.2 Hz, 1H), 7.76 (dt, *J* = 7.8, 1.1 Hz, 1H), 7.74–7.67 (m, 2H), 7.67–7.60 (m, 2H), 7.40 (t, *J* = 7.7 Hz, 1H), 7.18 (ddd, *J* = 7.7, 1.8, 1.0 Hz, 1H), 3.84 (s, 3H).

^{13}C-NMR: (126 MHz, CD_3CN) δ (ppm) = 149.7, 147.9, 145.2, 143.3, 142.0, 140.0, 139.8, 133.8, 132.9, 132.1, 130.6, 129.6, 129.4, 128.7, 128.0, 127.9, 120.9, 116.2, 39.9.

^{19}F-NMR: (282 MHz, CD_3CN) δ (ppm) = –62.05 (s, 3F), –72.96 (d, $^1J_{PF}$ = 706.3 Hz, 6F).

HRMS: ESI(+); *m/z* calculated for $C_{42}H_{30}F_{12}N_8P$ $[M–PF_6]^+$: 905.21, found: 905.2128 $[M–PF_6]^+$.

IR: $\tilde{\nu}$ (cm^{-1}) = 3162 (w), 1603 (w), 1483 (w), 1435 (w), 1395 (w), 1326 (m), 1172 (w), 1132 (m), 1074 (m), 1018 (w), 998 (w), 826 (s), 758 (w), 712 (w), 663 (w), 619 (w), 556 (s), 477 (w), 421 (w).

3. Synthesis of Racemic Iron Complex

rac-**FeNCCN**

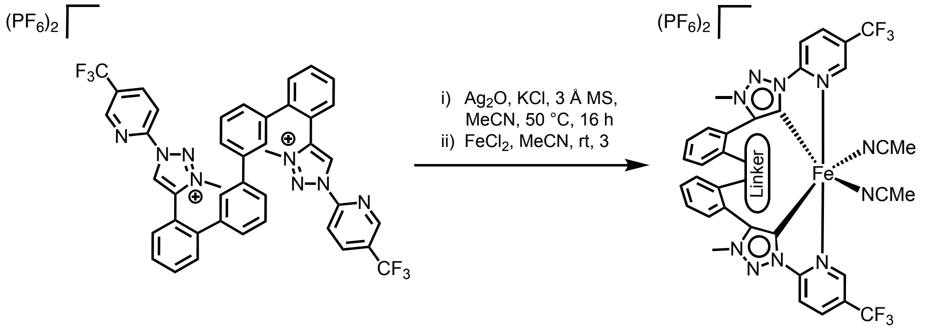

6 *rac*-**FeNCCN**

Following a modified procedure from the literature.[7] The ligand **6** (0.47 g, 0.45 mmol, 1.00 eq), Ag_2O (0.72 g, 3.13 mmol, 7.00 eq), KCl (0.33 g, 4.47 mmol, 10.00 eq) and molecular sieve (3 Å, 1 g per 1.0 mmol ligand) were suspended in dry and degassed MeCN (0.025 M based on the triazolium salt **6**) under an atmosphere of nitrogen. The Schlenk tube was sealed, and the mixture was stirred for 16 h under exclusion of light at 50 °C to generate the silver carbene intermediate. The grey suspension was allowed to cool to room temperature, diluted with MeCN, filtered over celite, and the solvent was removed under reduced pressure to obtain the crude Ag-carbene as an off-white solid. $FeCl_2$ (56.7 mg, 0.45 mmol, 1.00 eq) was placed in a SCHLENK tube under nitrogen atmosphere and the Ag-carbene was added together with dry and degassed MeCN (0.0125 M based on the triazolium salt), whereupon the mixture was stirred for 3 h at room temperature. The mixture was diluted with MeCN, filtered over celite, and the solvent was removed under reduced pressure. The residue was purified by flash column chromatography (silica gel, CH_2Cl_2/MeCN, 6:1 → 5:1) with a NH_4PF_6 pad on top of the column to ensure complete elution of the desired complex. After removing the solvent under reduced pressure, the product was dissolved in CH_2Cl_2/MeCN (50:1) and filtered over celite to remove excess of PF_6^--salts. The solvent of the filtrate was removed under reduced pressure to obtain the desired complex *rac*-**FeNCCN** (0.21 g, 0.18 mmol, 41%) as a purple solid.

^{1}H-NMR: (600 MHz, CD_3CN) δ (ppm) = 9.12–9.08 (m, 1H), 8.01 (dd, J = 8.6, 2.0 Hz, 1H), 7.77 (m, 2H), 7.64 (t, J = 7.7 Hz, 1H), 7.56 (m, 1H), 7.54–7.47 (m, 2H), 7.37 (dd, J = 7.6, 1.6 Hz, 1H), 7.33 (t, J = 7.4 Hz, 1H), 6.01 (t, J = 1.9 Hz, 1H), 3.01 (s, 3H), 1.96 (s, 3H).

^{13}C-NMR: (151 MHz, CD_3CN) δ (ppm) = 184.0, 157.2, 153.8, 151.8, 141.4, 140.3, 139.8, 138.4, 133.8, 132.3, 131.9, 130.7, 130.5, 129.4, 126.8, 125.8, 122.4, 113.4, 38.1.

^{19}F-NMR (282 MHz, CD_3CN) δ (ppm) = –62.33 (s, 3 F), –72.97 (d, $^{1}J_{PF}$ = 706.2 Hz, 6 F).

HRMS: ESI(+); *m/z* calculated for $C_{42}H_{26}F_6N_8Fe$ $[M-(PF_6)_2-(MeCN)_2]^{2+}$: 407.08, found: 407.0837 $[M-(PF_6)_2-(MeCN)_2]^{2+}$.

IR: ṽ (cm^{-1}) = 1619 (w), 1596 (w), 1502 (w), 1463 (w), 1422 (w), 1397 (w), 1322 (m), 1264 (w), 1186 (w), 1176 (w), 1146 (m), 1122 (w), 1079 (m), 1039 (w), 1022 (w), 990 (w), 892 (w), 876 (w), 831 (s), 786 (w), 769 (w), 755 (w), 742 (w), 724 (w), 707 (w), 699 (w), 675 (w), 657 (w), 627 (w), 615 (w), 556 (s), 505 (w), 497 (w), 438 (w).

4. Synthesis of Auxiliary Complexes

Λ-(*R*)-**FeAux** and Δ-(*R*)-**FeAux**

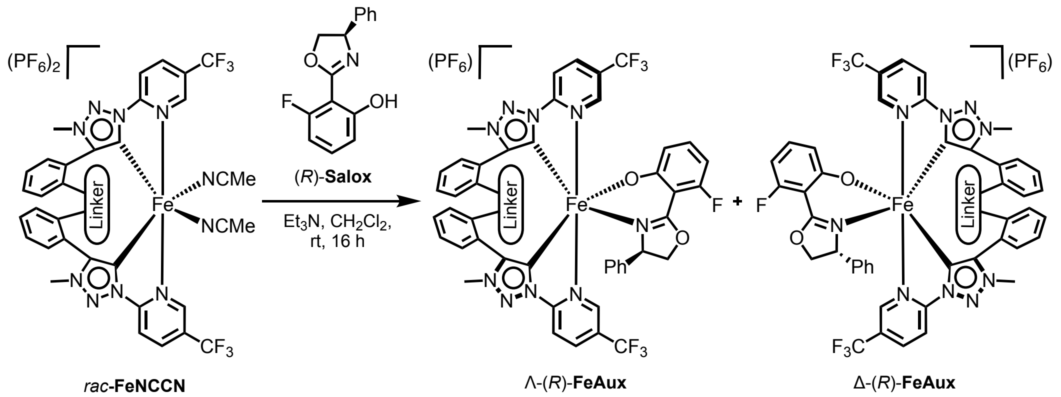

Following a procedure from the literature.[7] The racemic iron complex *rac*-**FeNCCN** (20.0 mg, 17.0 µmol, 1.00 eq) and the Salox auxiliary (4.60 mg, 18.0 µmol, 1.05 eq) were dissolved in dry CH_2Cl_2 (0.04 M based on the iron complex) under an atmosphere of nitrogen. Et_3N (3.50 µL, 25.0 µmol, 1.50 eq) was added, and the reaction mixture was stirred at room temperature for 16 h. The solvent was then removed under reduced pressure and the residue was purified by flash column chromatography (silica gel, CH_2Cl_2/MeCN, 100:1 → 75:1 → 50:1 → 20:1) to obtain Λ-(*R*)-**FeAux** (2.8 mg, 3.6 µmol, 13%) as black solid.

^{1}H-NMR: (500 MHz, CD_2Cl_2) δ (ppm) = 9.23 (s, 1H), 8.98 (s, 1H), 7.97 (s, 2H), 7.77 (d, *J* = 7.6 Hz, 1H), 7.78–7.74 (m, 1H), 7.68–7.65 (m, 1H), 7.63–7.55 (m, 5H), 7.54–7.40 (m, 5H), 7.39–7.31 (m, 3H), (t, *J* = 7.5 Hz, 2H), 7.29 (dd, *J* = 7.7, 1.3 Hz, 1H), 7.27 (s, 1H), 7.21 (t, *J* = 7.3 Hz, 2H), 6.92–6.87 (m, 1H), 6.59 (t, *J* = 7.5 Hz, 1H), 6.28 (d, *J* = 8.5 Hz, 1H), 6.17 (s, 1H), 5.67 (s, 1H), 4.75 (d, *J* = 7.6 Hz, 1H), 4.58 (dd, *J* = 9.3, 3.8 Hz, 1H), 4.24 (t, *J* = 9.4 Hz, 1H), 3.02 (s, 3H), 3.00 (s, 3H).

^{13}C-NMR: (126 MHz, CD_2Cl_2) δ (ppm) = 165.8, 163.3, 161.3, 158.4, 157.7, 154.0, 152.0, 140.8, 140.6, 140.1, 139.8, 139.6, 139.5, 135.0, 134.7, 134.0, 131.8, 131.6, 131.3, 131.2, 130.8, 130.4, 130.0, 129.9, 129.5, 129.3, 129.1, 128.7, 127.4, 126.9, 126.6, 126.5, 126.2, 126.2, 126.1, 125.8, 124.8, 123.9, 123.5, 121.8, 111.7, 77.0, 37.5, 37.3.

^{19}F-NMR: (282 MHz, CD_2Cl_2) δ (ppm) = –61.97 (s, 3F), –62.67 (s, 3F), –73.45 (d, $^{1}J_{PF}$ = 710.5 Hz,6F), –110.44 (s, 1F).

HRMS: ESI(+); *m/z* calculated for $C_{57}H_{39}F_7FeN_9O_2$ [M–(PF_6)]$^+$: 1070.25, found: 1070.2437 [M–(PF_6)]$^+$.

IR: ṽ (cm^{-1}) = 3067 (w), 2955 (w), 2924 (w), 2854 (w), 1660 (w), 1619 (m), 1580 (w), 1538 (w), 1492 (w), 1447 (m), 1386 (w), 1322 (s), 1260 (w), 1232 (w), 1172 (w), 1139 (m), 1078 (w), 1039 (w), 1018 (w), 980 (w), 951 (w), 839 (s), 797 (w), 767 (w), 705 (w), 656 (w), 627 (w), 558 (m), 532 (w), 497 (w).

Notes: The ^{19}F-NMR spectrum shows some impurities of free auxiliary due to the decomposition of this less stable diastereomer on the column.

The other diastereomer Δ-(*R*)-**FeAux** (10.4 mg, 8.4 µmol, 49%) was obtained as a black solid.

^{1}H-NMR: (600 MHz, CD_2Cl_2) δ (ppm) = 8.76 (s, 1H), 8.60 (s, 1H), 7.71–7.65 (m, 2H), 7.63–7.24 (m, 7H), 7.45–7.36 (m, 5H), 7.33 (q, *J* = 8.3 Hz, 2H), 7.24 (t, *J* = 7.4 Hz, 1H), 7.10 (s, 1H), 6.92–6.84 (m, 2H), 6.77 (s, 2H), 6.53 (s, 1H), 6.25 (d, *J* = 7.4 Hz, 2H), 6.02 – 5.92 (m, 2H), 5.70 (s, 1H), 5.53 (s, 1H), 5.02 (t, *J* = 8.9 Hz, 1H), 4.39 (d, *J* = 9.0 Hz, 1H), 3.08 (s, 3H), 2.95 (s, 3H).

^{13}C-NMR: (151 MHz, CD_2Cl_2) δ (ppm) = 158.5, 156.6, 151.2, 150.8, 150.6, 141.6, 140.7, 139.9, 139.6, 139.4, 134.6, 133.7, 131.8, 131.8, 131.7, 131.3, 131.2, 130.9, 130.1, 129.4, 129.3, 129.2, 128.7, 128.6, 128.4, 126.3, 126.2, 126.0, 125.9, 125.8, 125.5, 125.4, 124.5, 124.1, 123.7, 121.9, 112.5, 111.5, 75.7, 37.8, 37.2.

^{19}F-NMR: (282 MHz, CD_2Cl_2) δ (ppm) = –62.38 (s, 3F), –62.59 (s, 3F), –73.40 (d, $^1J_{PF}$ = 710.5 Hz, 6F), –106.40 (s, 1F).

HRMS: ESI(+); *m/z* calculated for $C_{57}H_{39}F_7FeN_9O_2$ [M–(PF_6)]$^+$: 1070.25, found: 1070.2474 [M–(PF_6)]$^+$.

IR: ṽ (cm^{-1}) = 3079 (w), 1616 (m), 1578 (m), 1530 (w), 1493 (w), 1448 (m), 1386 (w), 1318 (s), 1302 (w), 1253 (w), 1231 (w), 1172 (w), 1136 (m), 1102 (w), 1078 (m), 1062 (w), 1040 (w), 1019 (w), 980 (w), 954 (w), 924 (w), 876 (w), 831 (s), 796 (w), 755 (m), 701 (m), 675 (w), 655 (w), 627 (w), 600 (w), 582 (w), 557 (m), 534 (w), 498 (w), 457 (w).

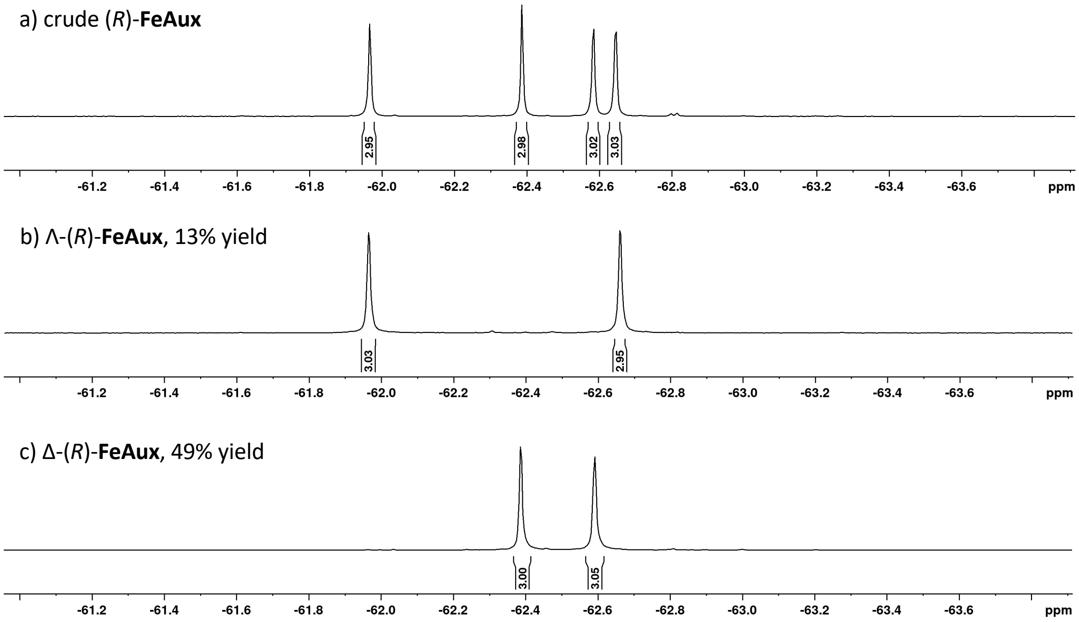

Figure S1: Enlarged excerpt of the ^{19}F-NMR spectra (282 MHz, CD_2Cl_2, 25 °C) of the auxiliary complexes showing the initial formation of both diastereomers during synthesis.

5. Cleavage of the Chiral Auxiliary

General Procedure A: Cleavage of Salox Auxiliary

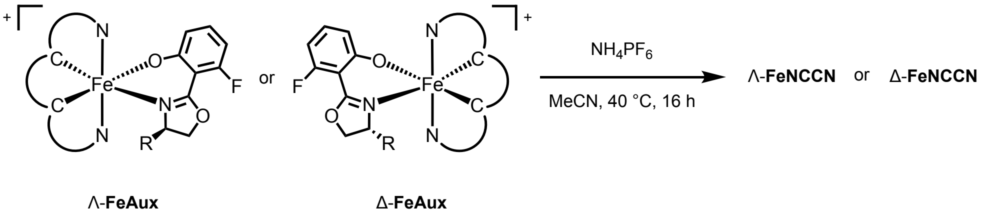

Following a modified procedure from the literature.[8] The single diastereomers of the auxiliary complexes (1.00 eq) were dissolved in dry MeCN (0.02 M based on the auxiliary complex) and an excess of NH_4PF_6 (10.00 eq) was added and the mixture was stirred at 40 °C for 16 h. Afterwards, the solvent was removed under reduced pressure and Et_2O was added. The precipitated solids were transferred to a celite pad and washed with Et_2O several times to remove any residues of free auxiliary. The complex was then eluted with CH_2Cl_2/MeCN, 30:1 to afford the corresponding Λ- or Δ-iron complexes after the solvent had been removed under reduced pressure.

Λ-**FeNCCN**

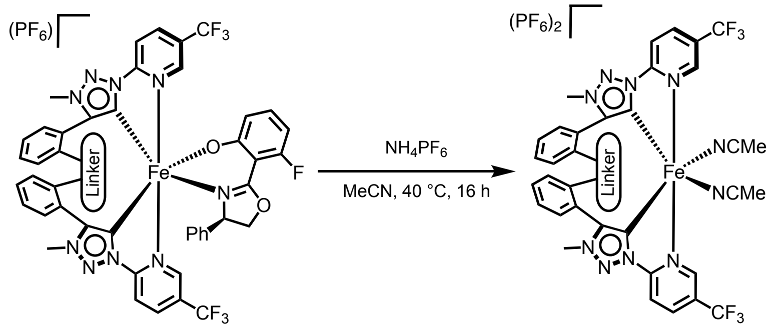

Λ-(*R*)-**FeAux** Λ-**FeNCCN**

Following general procedure [**A**], Λ-**FeNCCN** (1.4 mg, 1.18 µmol, 94%) was obtained as a purple solid from the corresponding complex Λ-(*R*)-**FeAux** (1.55 mg, 1.27 µmol). The spectroscopic data of the enantiopure complex were in accordance with *rac*-**FeNCCN**.

CD (MeCN, 0.25 mM): λ, nm (Δε, $M^{-1}cm^{-1}$) 230 (+81), 248 (−17), 270 (+56), 303 (−9), 344 (+5), 420 (−9), 456 (+5), 500 (−7), 574 (+4).

Δ-**FeNCCN**

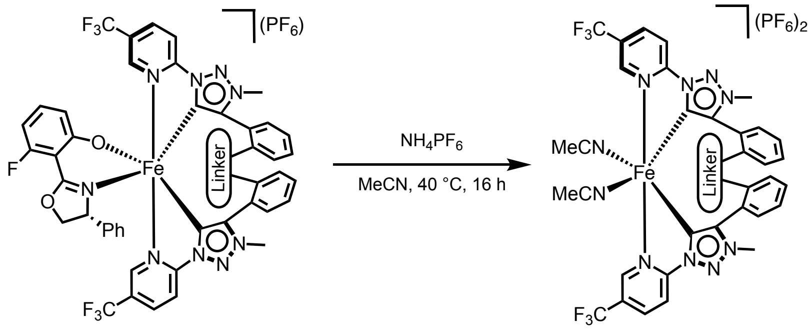

Δ-(*R*)-**FeAux** Δ-**FeNCCN**

Following general procedure [**A**], Δ-**FeNCCN** (72.5 mg, 61.1 µmol, 94%) was obtained as a purple solid from the corresponding complex Δ-(*R*)-**FeAux** (80.0 mg, 65.8 µmol). The spectroscopic data of the enantiopure complex were in accordance with *rac*-**FeNCCN**.

CD (MeCN, 0.25 mM): λ, nm (Δε, $M^{-1}cm^{-1}$) 230 (−86), 248 (+18), 270 (−61), 303 (+10), 344 (−6), 420 (+10), 456 (−5), 500 (+8), 574 (−4).

6. Determination of Enantiomeric Excess of Λ- and Δ-FeNCCN

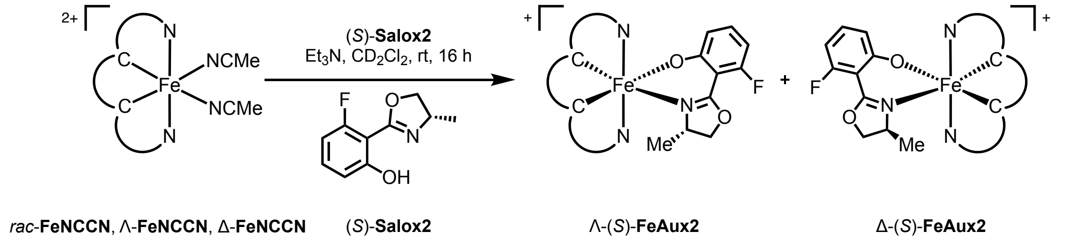

Following a procedure from the literature.[9] The iron complexes (1.00 eq) and the auxiliary (*S*)-**Salox2** (4.00 eq) were dissolved in CD_2Cl_2 (0.004 M based on the iron complex), followed by the addition of Et_3N (8.00 eq). The mixture was placed in an NMR tube equipped with a magnetic stirring bar and stirred at room temperature for 16 h. A color change could be observed that correlated with the corresponding auxiliary complexes. The sample was then directly subjected to ^{19}F-NMR with 1000 scans, showing the diastereomeric ratio between Λ-(*S*)-**FeAux2** and Δ-(*S*)-**FeAux2.**

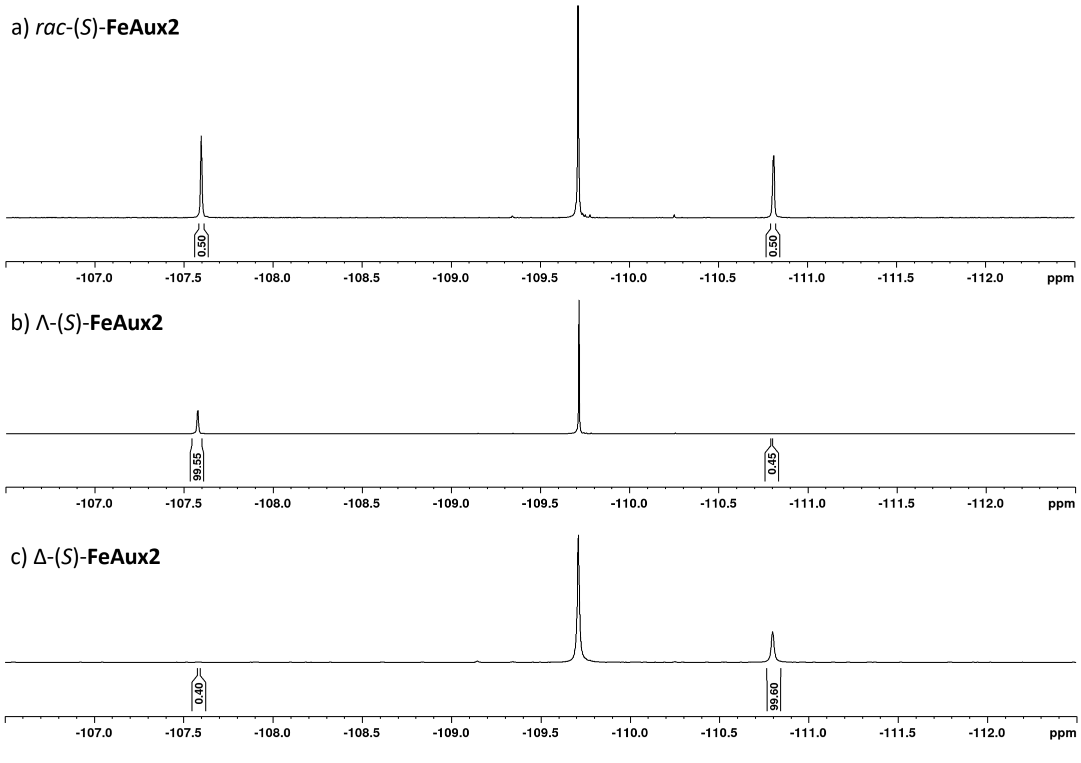

Figure S2: Enlarged excerpt of the ^{19}F-NMR spectra (282 MHz, CD_2Cl_2, 25 °C) with 1000 scans of the auxiliary complexes after recoordination of the auxiliary (*S*)-**Salox2**.

7. Stability Experiments

Procedure:

A sample of the racemic complex in the corresponding solvent (CD_3CN or CD_2Cl_2) was prepared in an NMR tube and examined by ^{1}H-NMR after various incubation times. The samples were stored at room temperature under air. Figure S3 shows the overlayed spectra in CD_3CN, while the measured spectra in CD_2Cl_2 are shown in Figure S4.

Results:

The racemic complex *rac*-**FeNCCN** exhibits high stability in CD_3CN with no indications of decomposition even after 21 days of storage at room temperature under air (Figure S3c), as shown by comparisons of the ^{1}H-NMR spectra with the original sample and the measurement after 7 days (Figure S3a+b). When comparing the stability of the complex in CD_2Cl_2, differences began to emerge immediately after 2 days, when slight decomposition was observed, as indicated by the signal of the methyl group of the free triazolium ligand **6** at 3.6 ppm in the ^{1}H NMR (Figure S4c). This continued in subsequent measurements after 3 days and 4 days of storage at room temperature under air (Figure S4d+e) until even more recognizable decomposition after 7 days (Figure S4f).

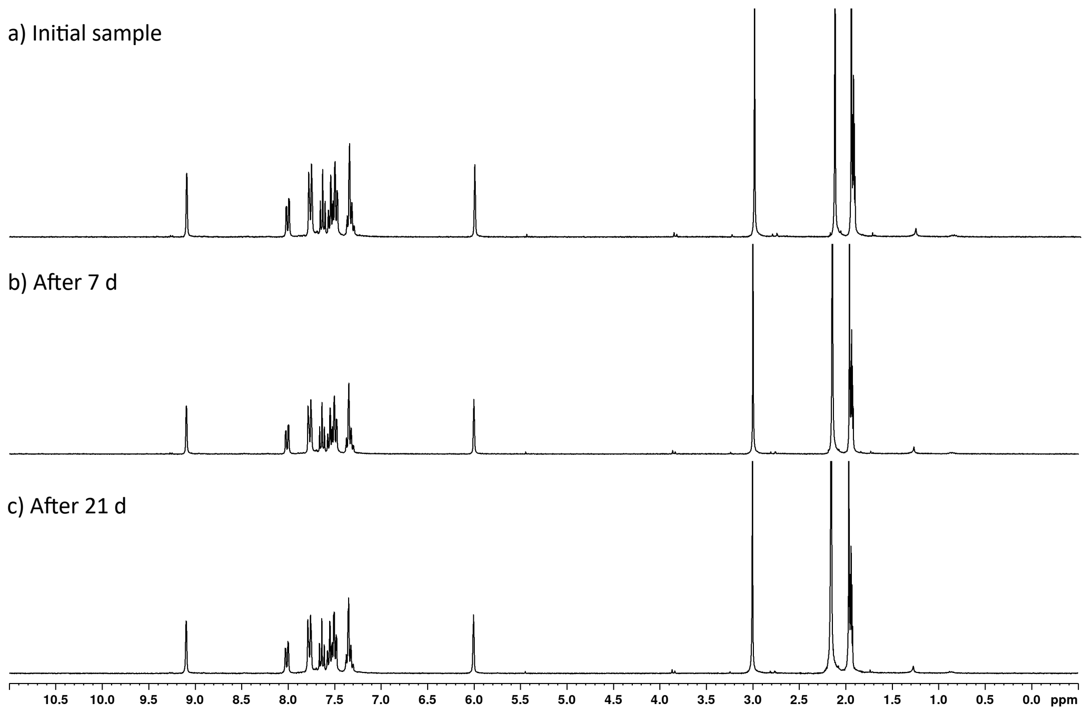

Figure S3: ^{1}H-NMR spectra (300 MHz, CD_3CN, 25 °C) of *rac*-**FeNCCN** after different periods of storage in CD_3CN.

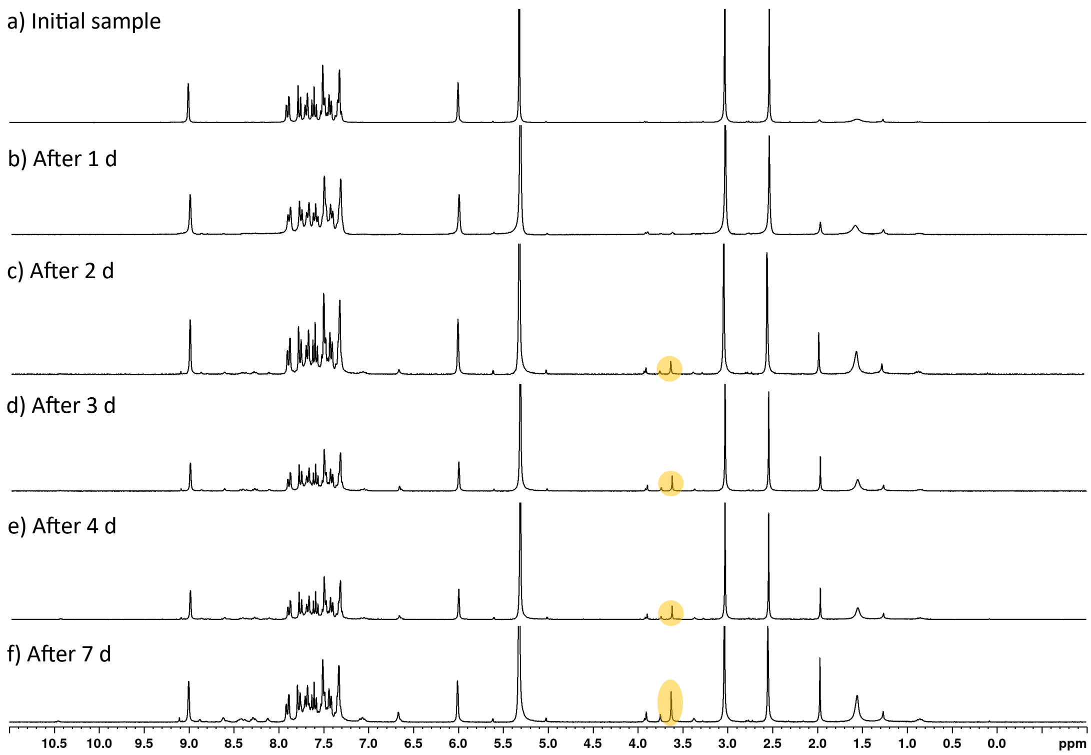

Figure S4: ^{1}H-NMR spectra (300 MHz, CD_2Cl, 25 °C) of *rac*-**FeNCCN** after different periods of storage in CD_2Cl_2**.**

8. Catalysis

C(*sp*^{3})-H amidation of Urea Derivative 7

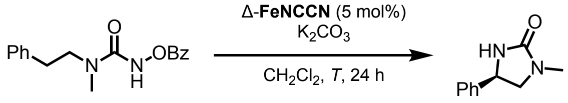

7 **8**

Following a literature known procedure.[7] The iron catalyst (5 mol%), K_2CO_3 (13.9 mg, 0.10 mmol, 3.00 eq) and the substrate **7** (10.0 mg, 0.03 mmol, 1.00 eq) were placed in a Schlenk tube under an atmosphere of nitrogen. Dry and degassed CH_2Cl_2 (0.05 M) was added, and the reaction mixture was stirred for the indicated time at the indicated temperature. According to that, a saturated aqueous NH_4Cl-solution (1.0 mL) was added the mixture was extracted with EtOAc (3 ×). The combined organic phases were dried over $MgSO_4$, filtered and the solvent was removed under reduced pressure and the yield was determined by ^{1}H-NMR analysis of the crude product with TMB as internal standard. The enantiomeric excess of the crude product was determined by HPLC analysis on a chiral stationary phase. The spectroscopic data are in accordance with the literature.[10] The absolute configuration of the product was determined by comparison of the HPLC traces with the literature.[10]

TLC: R_f = 0.17 (*n*-pentane/EtOAc 2:1).

^{1}H-NMR (300 MHz, $CDCl_3$) δ (ppm) = 7.35–7.13 (m, 5H), 5.42 (s, 1H), 4.69 (s, 1H), 4.04 (q, J = 7.1 Hz, 1H), 3.55–3.44 (m, 1H), 2.79 (s, 3H).

HPLC: Daicel Chiralcel® IA column, 250 x 4.6 mm, absorbance at 220 nm, *n*-hexane/iPrOH 95:5, isocratic flow, flow rate 1.0 mL/min, 30 °C, t_r (minor) = 23.68 min, t_r (major) = 29.13 min.

Cannizzaro Reaction of Phenylglyoxal (9)

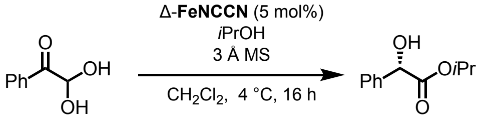

9 **10**

Following a literature known procedure.[8] The iron catalyst (5 mol%), molecular sieve (3 Å, 5 mg per 0.01 mmol substrate) and phenylglyoxal (**9**) (7.6 mg, 0.05 mmol, 1.00 eq) were suspended in dry and degassed CH_2Cl_2 (0.05 M) under an atmosphere of nitrogen in a Schlenk tube. Then *i*PrOH (37.5 µL, 0.50 mmol, 10.0 eq) was added and the mixture was stirred for 16 h at the indicated temperature. According to that Et_2O was added and the reaction mixture was filtered over a short plug of silica gel to remove the catalyst. The solvent was removed under reduced pressure and the yield was determined by ^{1}H-NMR analysis of the crude product with TMB as internal standard. The enantiomeric excess of the crude product was determined by HPLC analysis on a chiral stationary phase. The spectroscopic data are in accordance with the literature.[8] The absolute configuration of the product was determined by comparison of the HPLC traces with the literature.[8]

TLC: R_f = 0.41 (*n*-pentane/EtOAc 3:1).

^{1}H-NMR (300 MHz, $CDCl_3$) δ (ppm) = 7.47–7.25 (m, 5H), 5.08 (m, 2H), 3.52 (d, *J* = 5.9 Hz, 1H), 1.28 (d, *J* = 6.3 Hz, 3H), 1.11 (d, *J* = 6.2 Hz, 3H)

HPLC: Daicel Chiralcel® OD-H column, 250 x 4.6 mm, absorbance at 210 nm, *n*-hexane/iPrOH 90:10, isocratic flow, flow rate 1.0 mL/min, 25 °C, t_r (minor) = 5.63 min, t_r (major) = 9.68 min.

9. NMR Spectra

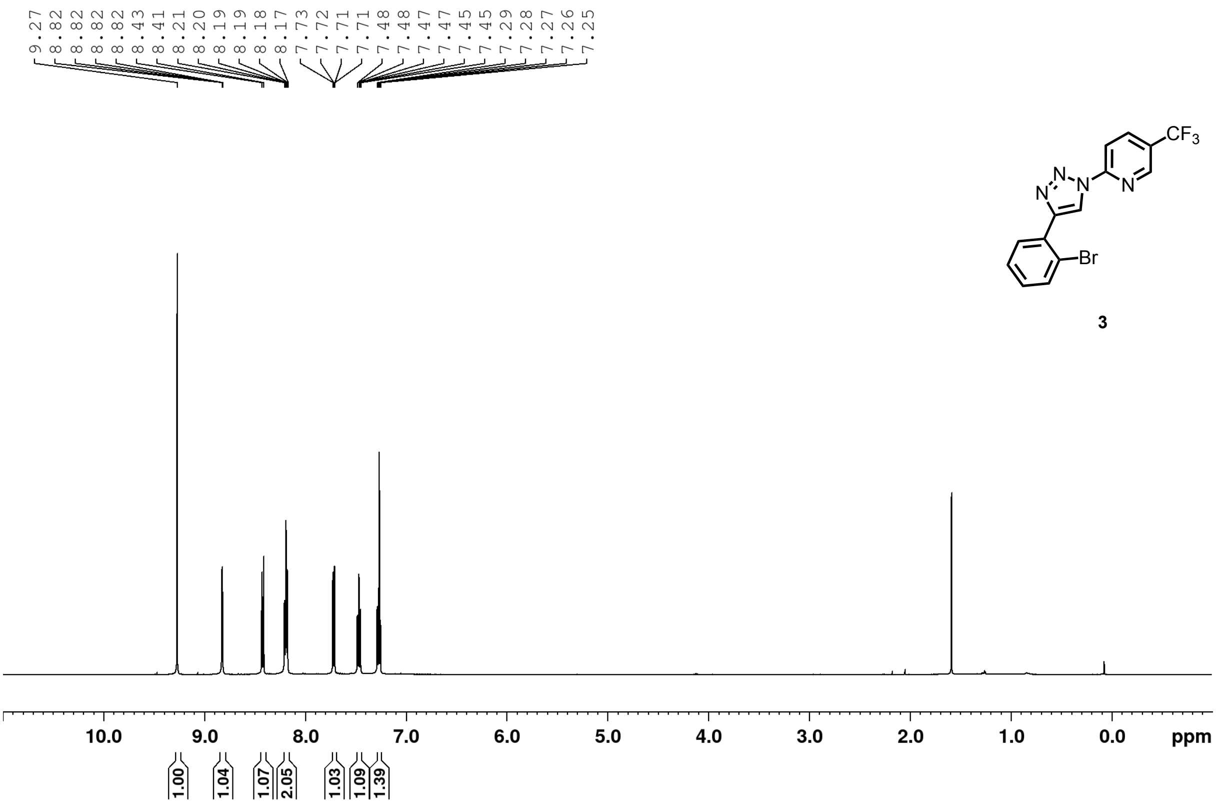

Figure S5: ^{1}H-NMR spectrum of **3** (600 MHz, $CDCl_3$, 25 °C).

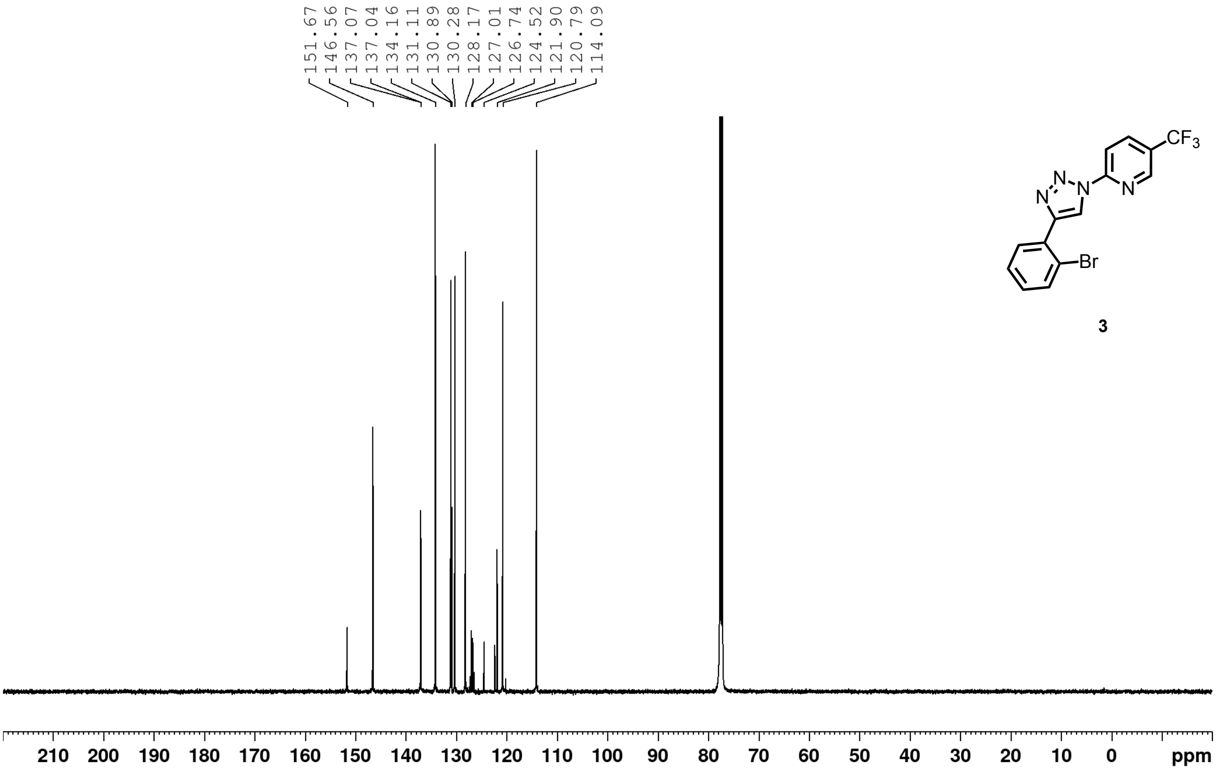

Figure S6: ^{13}C-NMR spectrum of **3** (151 MHz, $CDCl_3$, 25 °C).

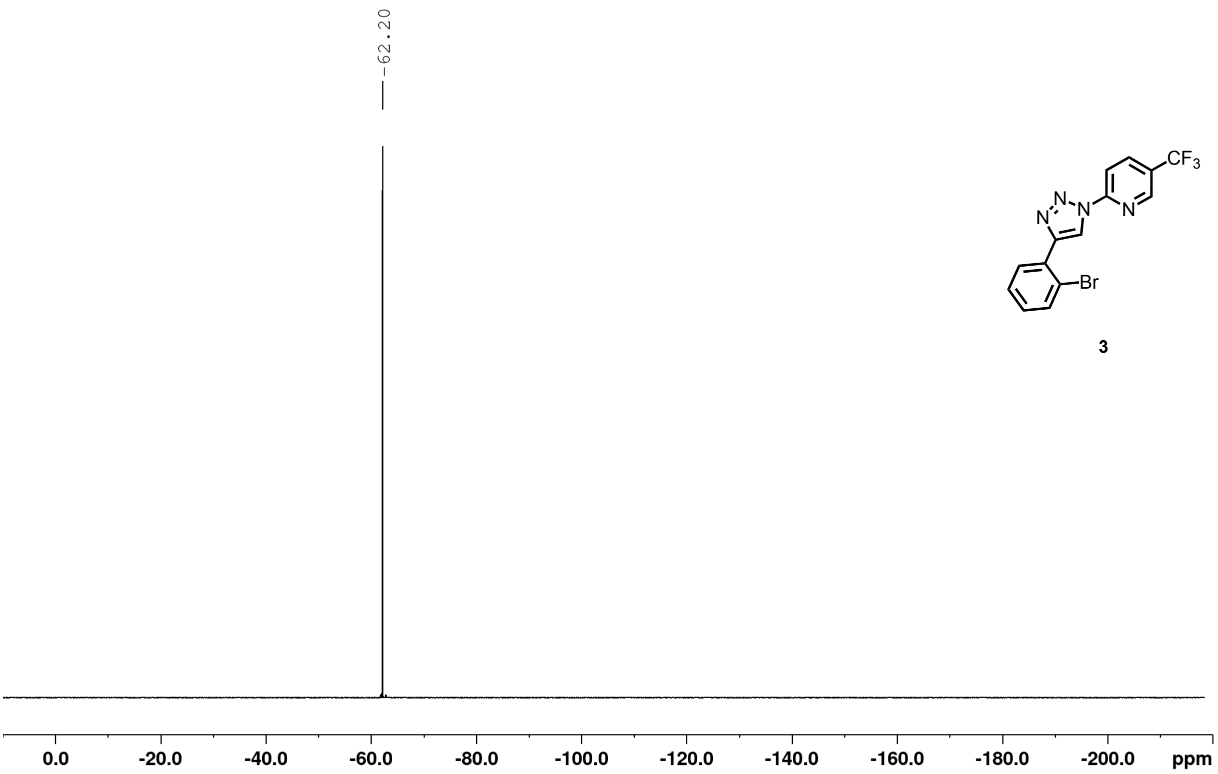

Figure S7: ^{19}F-NMR spectrum of **3** (282 MHz, $CDCl_3$, 25 °C).

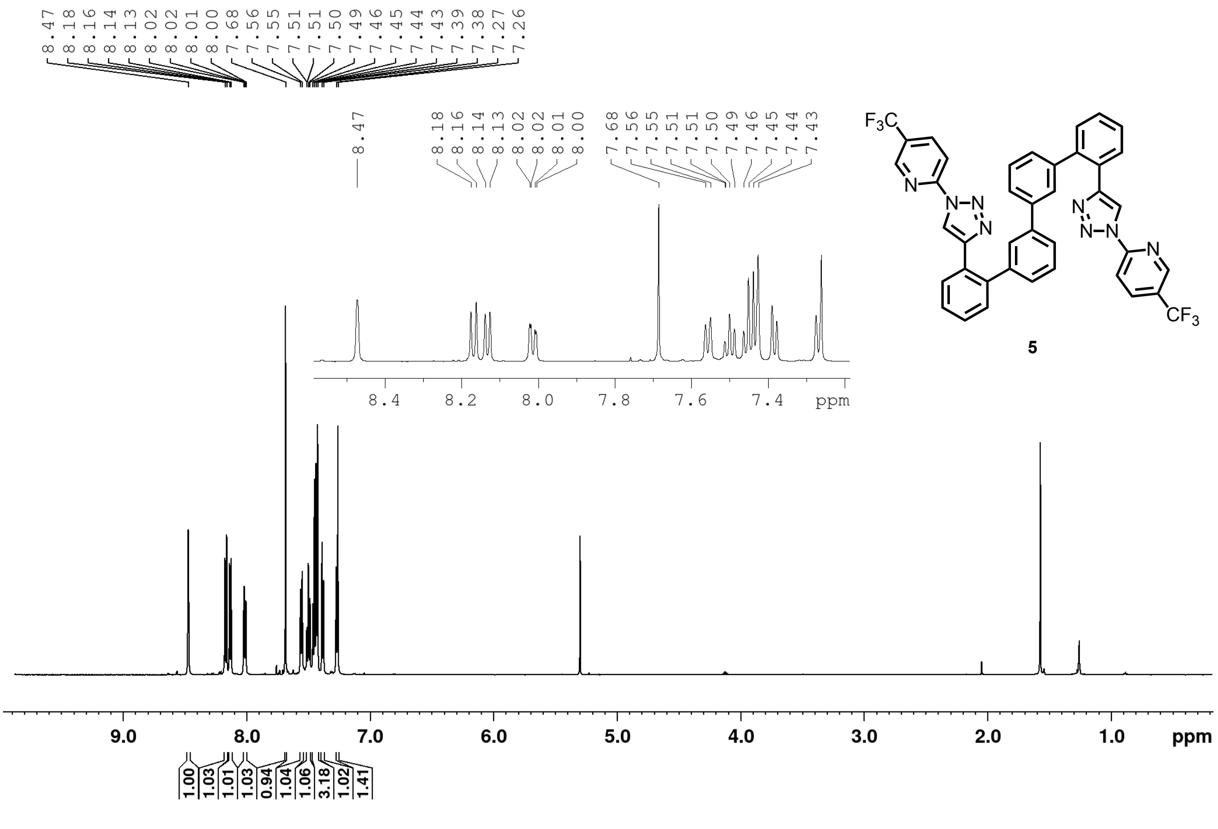

Figure S8: ^{1}H-NMR spectrum of **5** (600 MHz, $CDCl_3$, 25 °C).

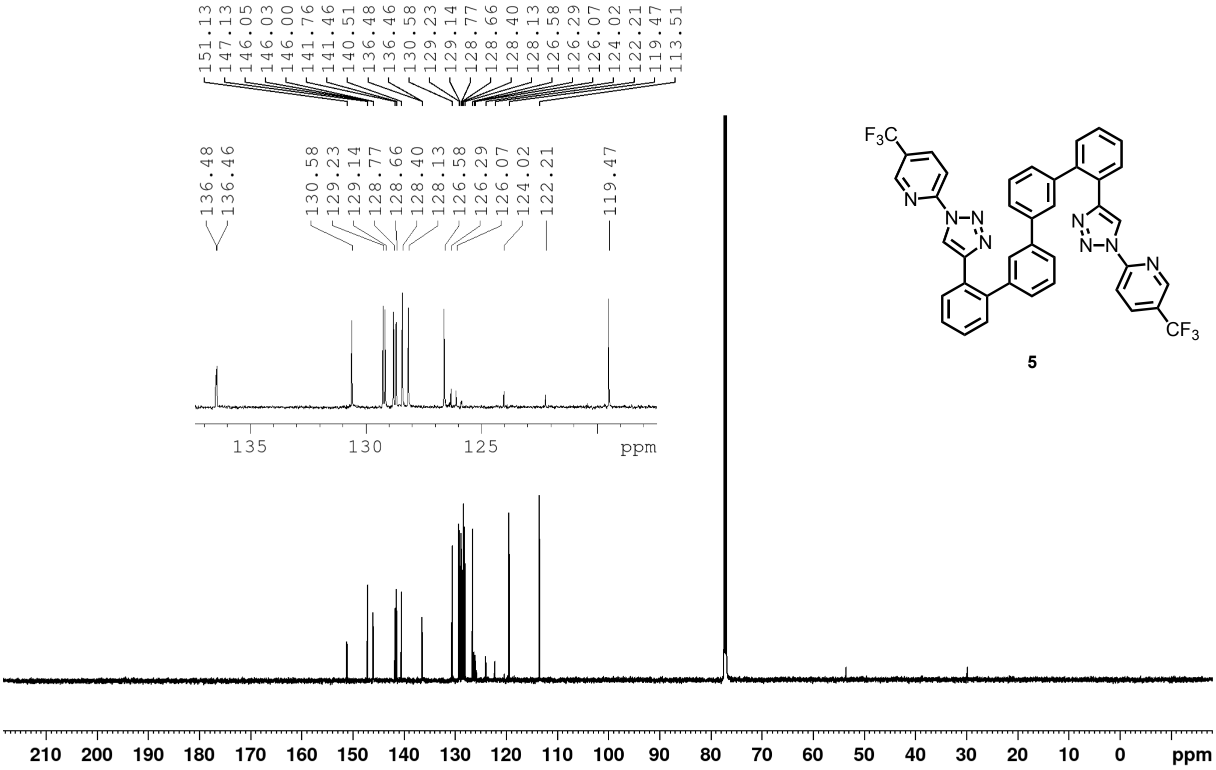

Figure S9: ^{13}C-NMR spectrum of **5** (151 MHz, $CDCl_3$, 25 °C).

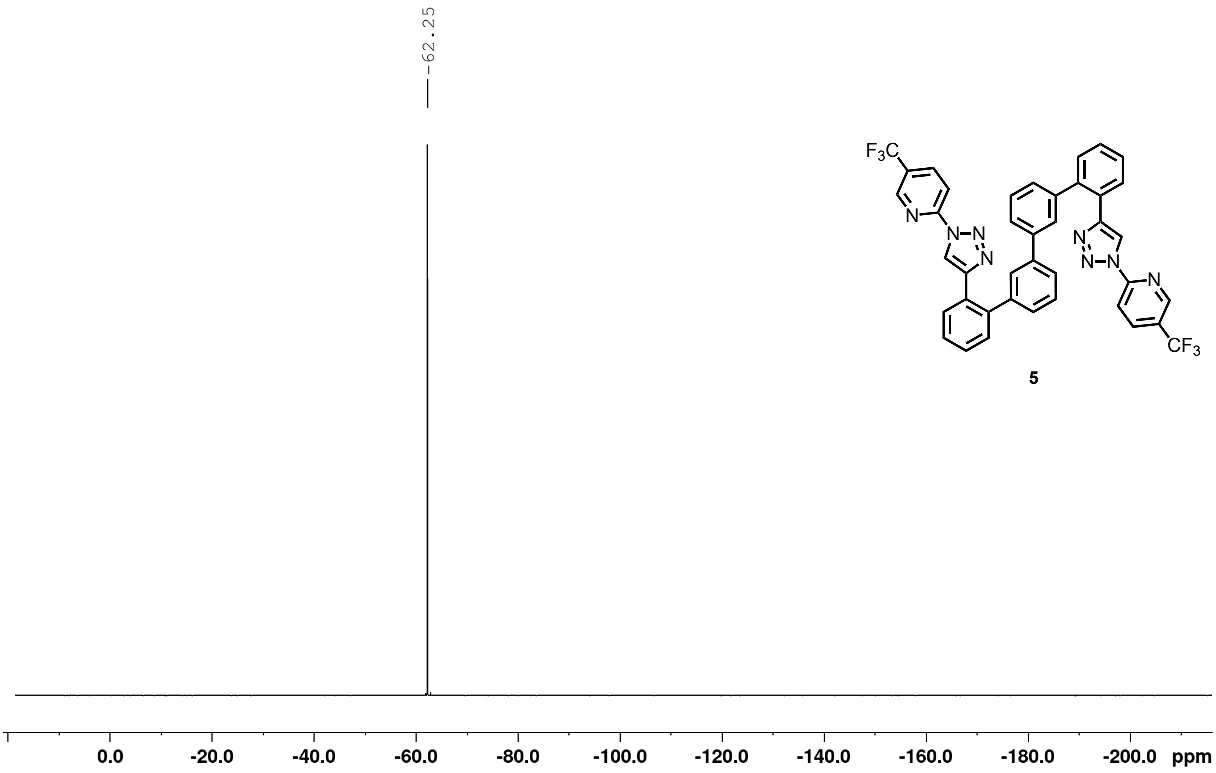

Figure S10: ^{19}F-NMR spectrum of **5** (282 MHz, $CDCl_3$, 25 °C).

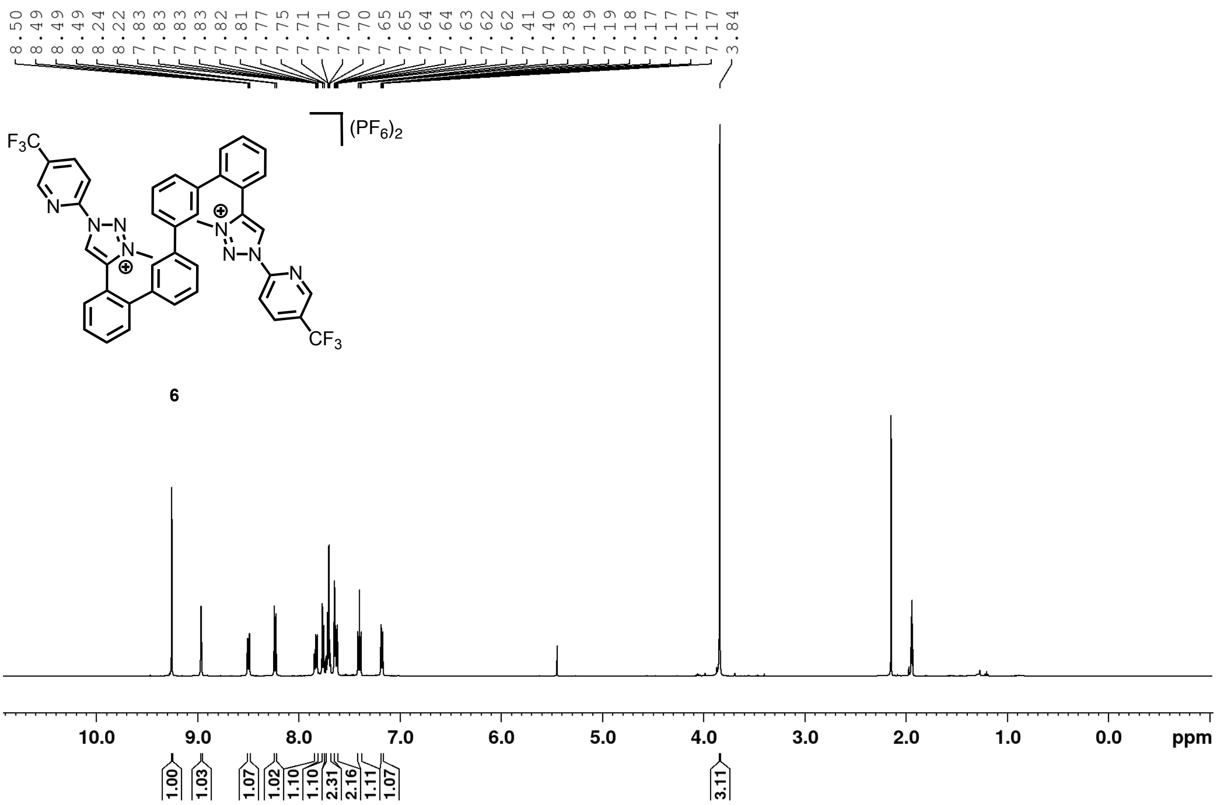

Figure S11: ^{1}H-NMR spectrum of **6** (500 MHz, CD_3CN, 25 °C).

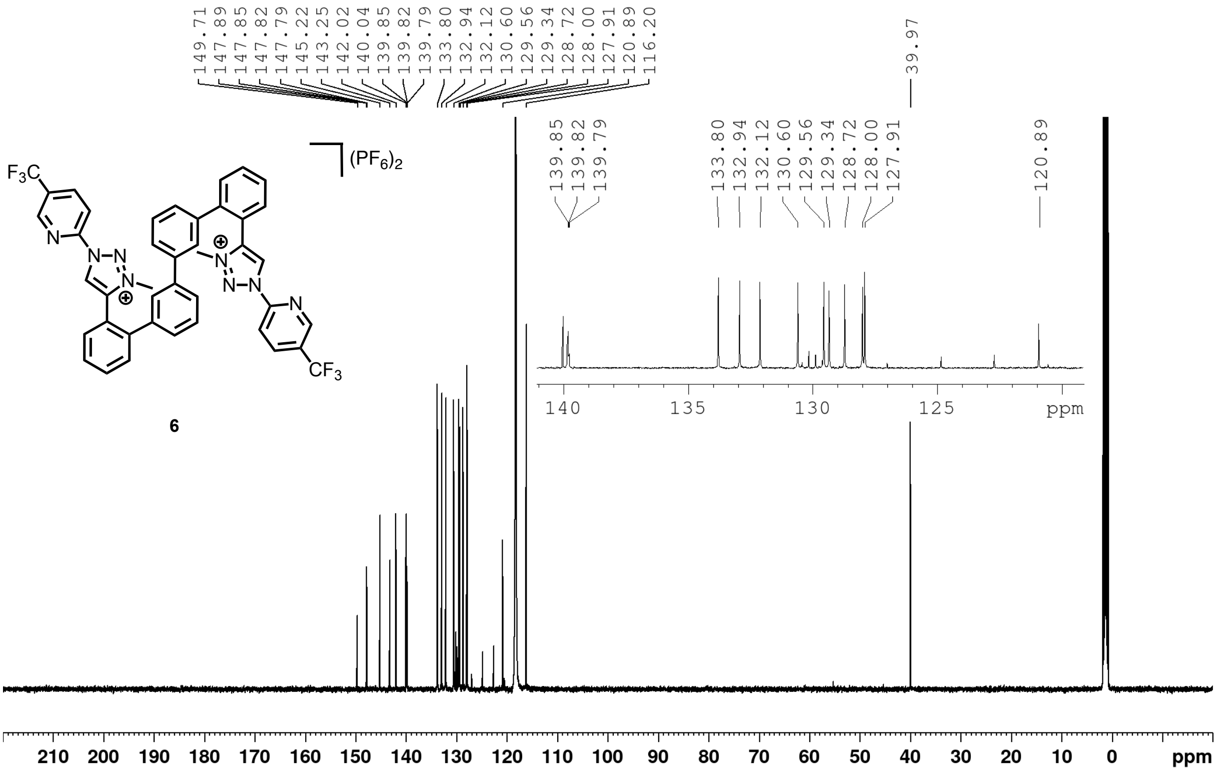

Figure S12: ^{13}C-NMR spectrum of **6** (126 MHz, CD_3CN, 25 °C).

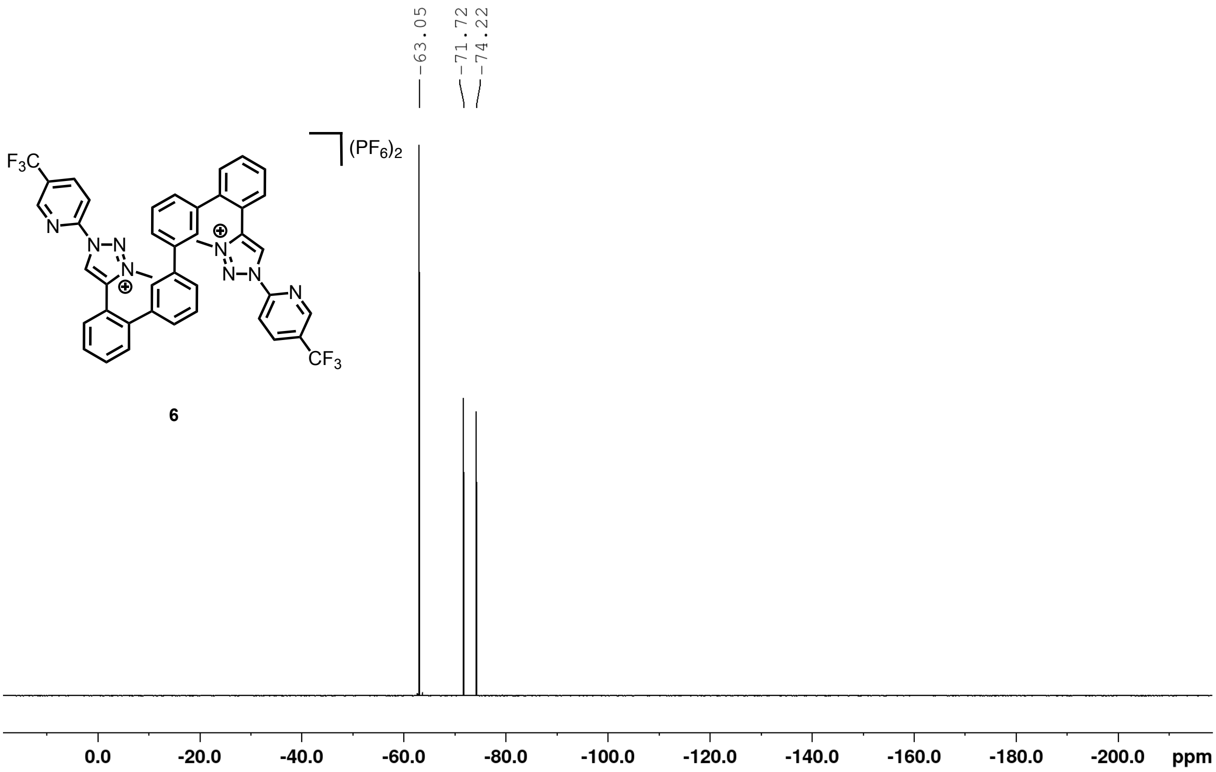

Figure S13: ^{19}F-NMR spectrum of **6** (282 MHz, CD_3CN, 25 °C).

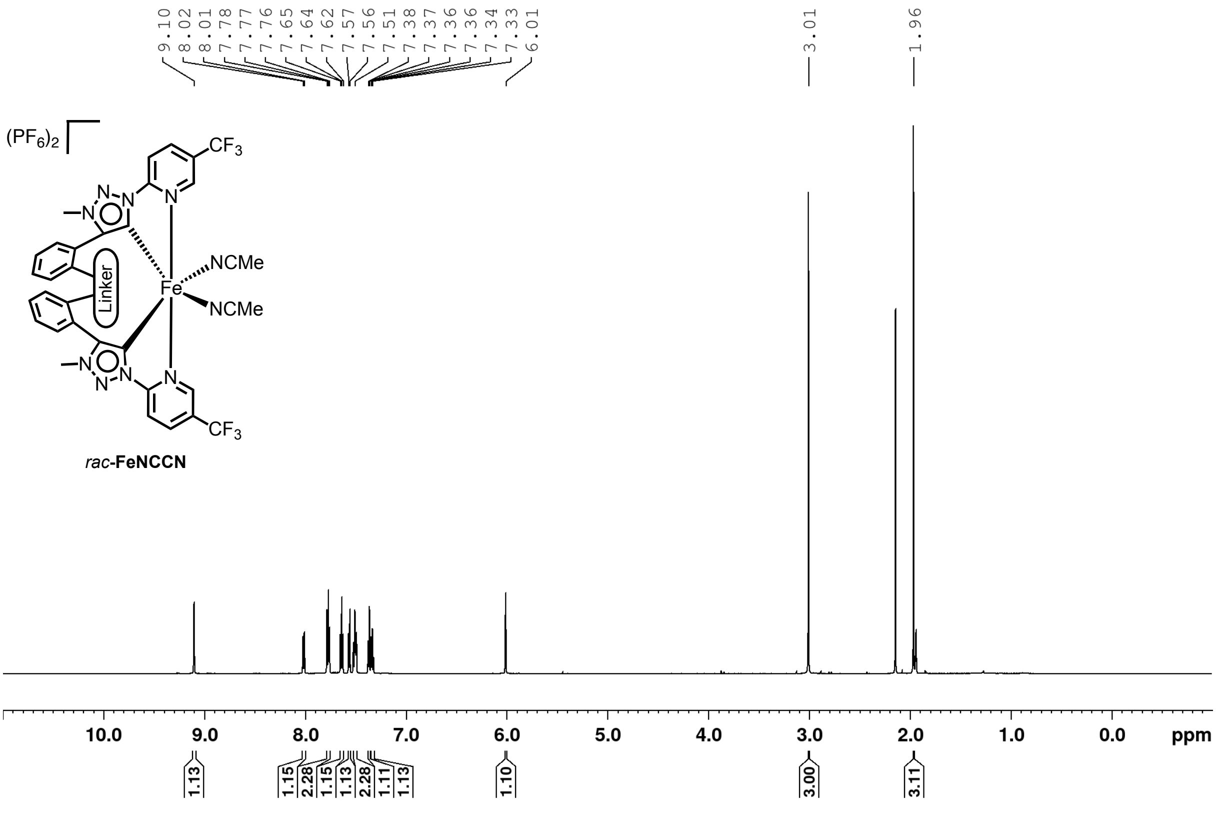

Figure S14: ^{1}H-NMR spectrum of *rac*-**FeNCCN** (600 MHz, CD_3CN, 25 °C).

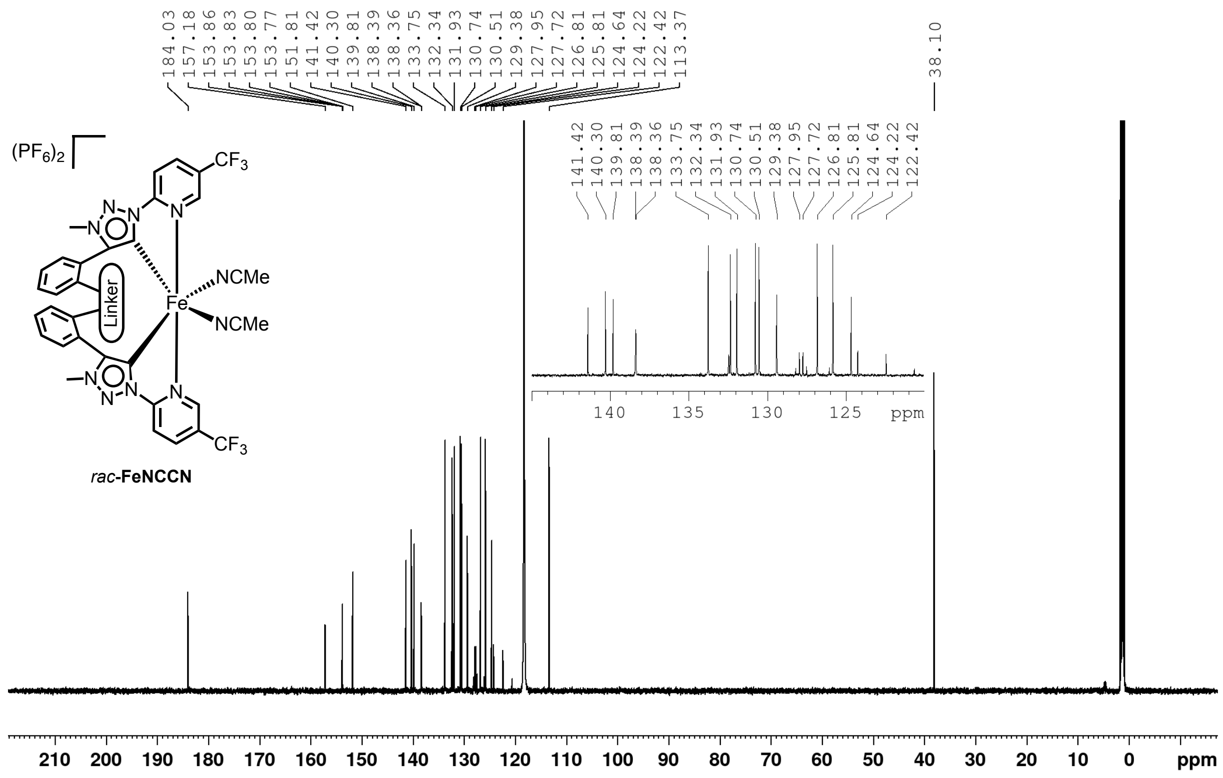

Figure S15: ^{13}C-NMR spectrum of *rac*-**FeNCCN** (151 MHz, CD_3CN, 25 °C).

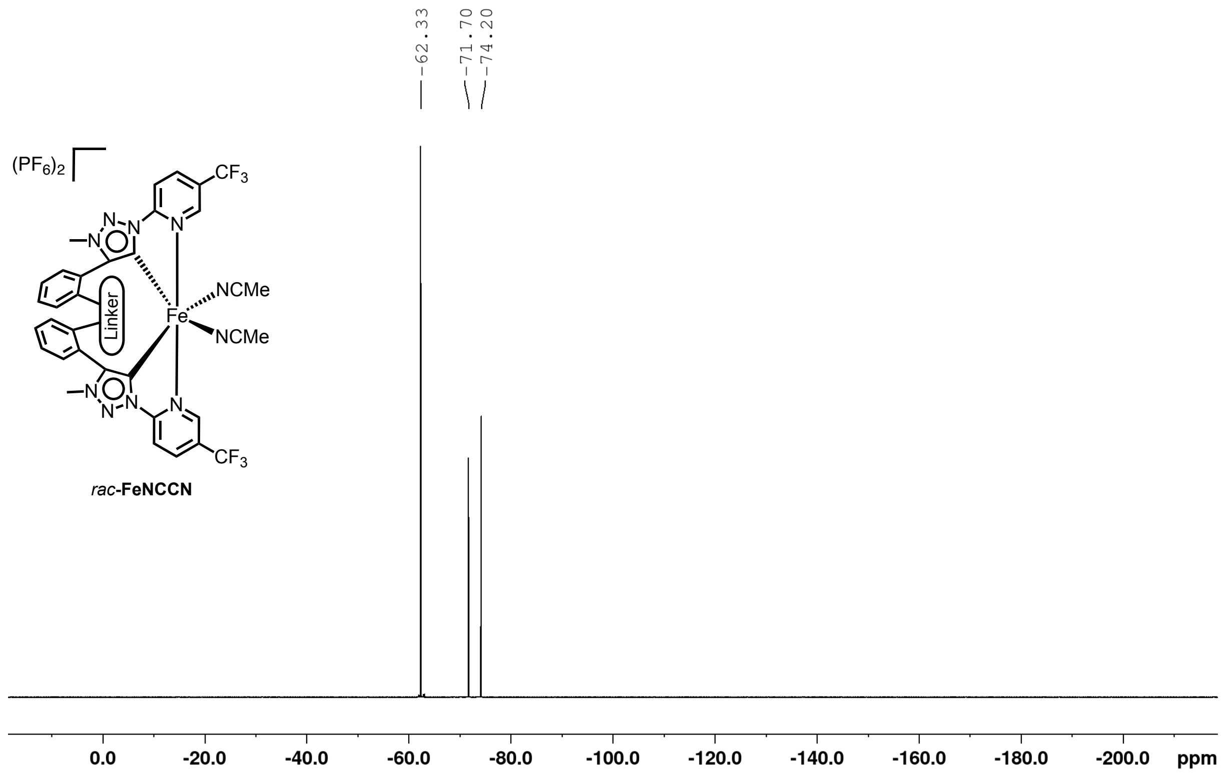

Figure S16: ^{19}F-NMR spectrum of *rac*-**FeNCCN** (282 MHz, CD_3CN, 25 °C).

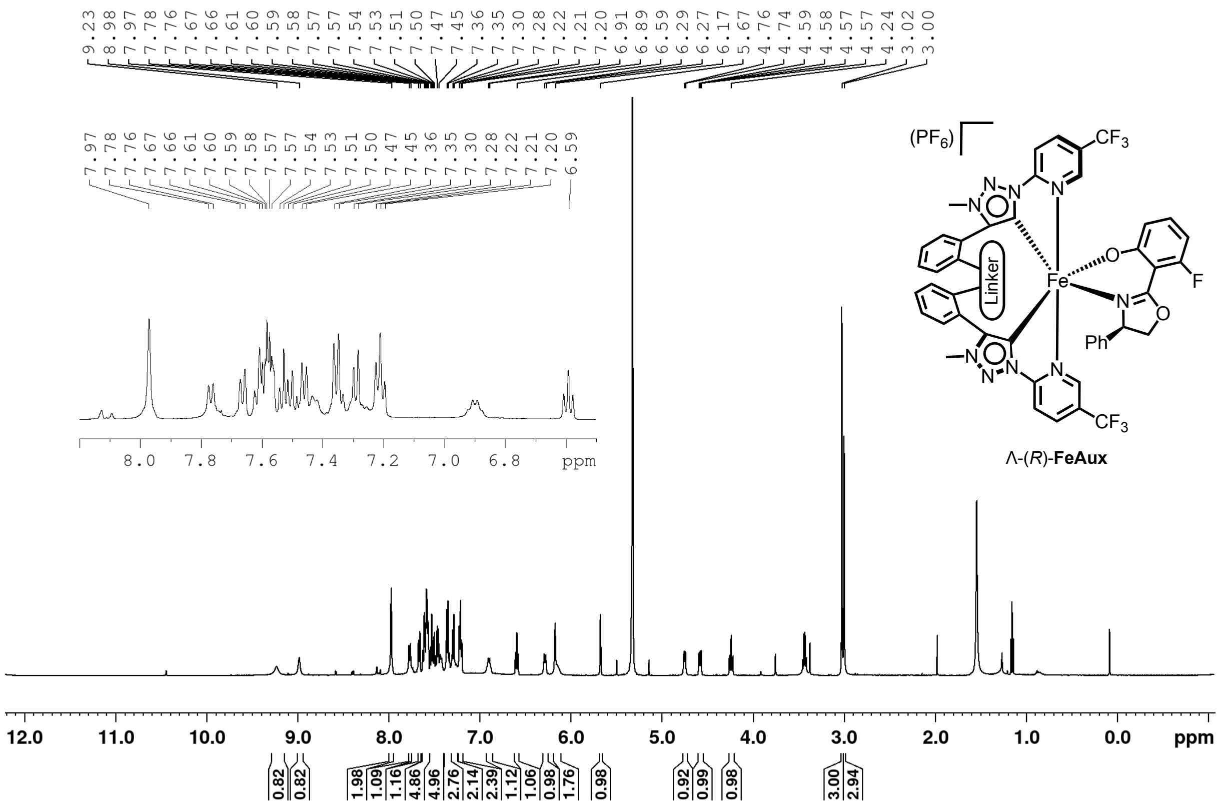

Figure S17: ^{1}H-NMR spectrum of Λ-(*R*)-**FeAux** (600 MHz, CD_2Cl_2, 25 °C).

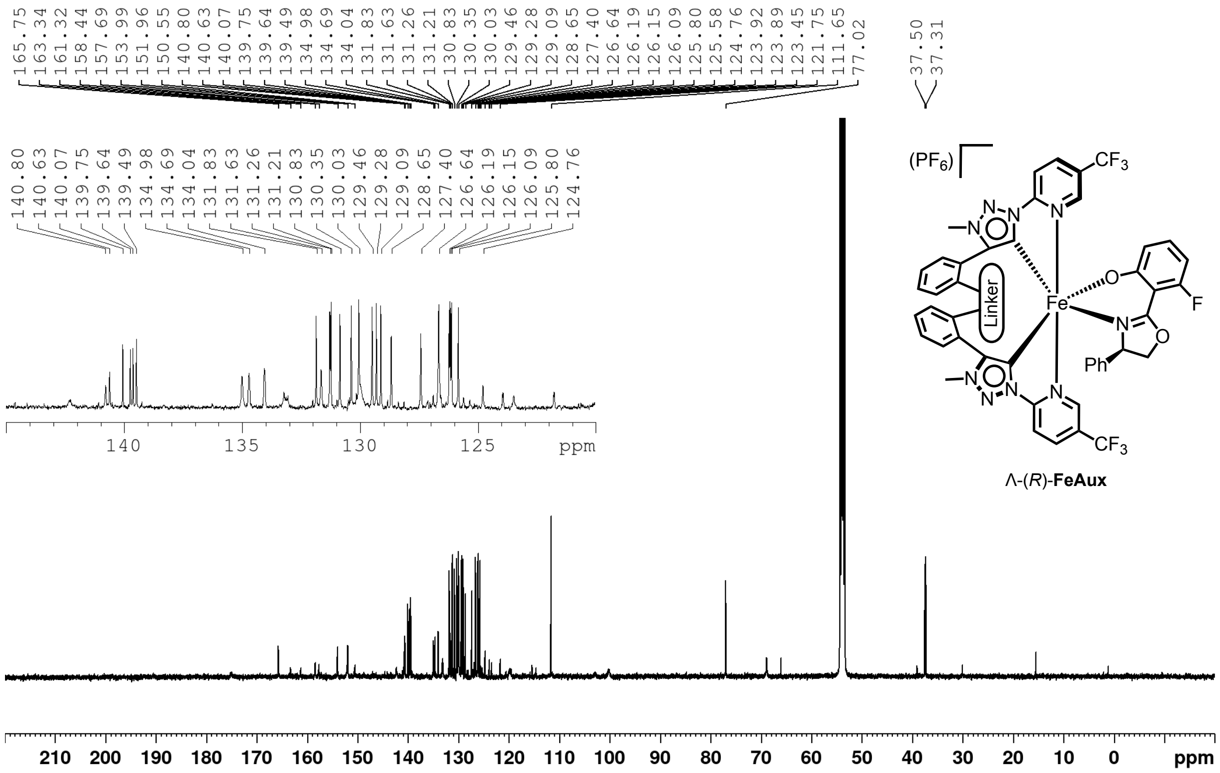

Figure S18: ^{13}C-NMR spectrum of Λ-(*R*)-**FeAux** (126 MHz, CD_2Cl_2, 25 °C).

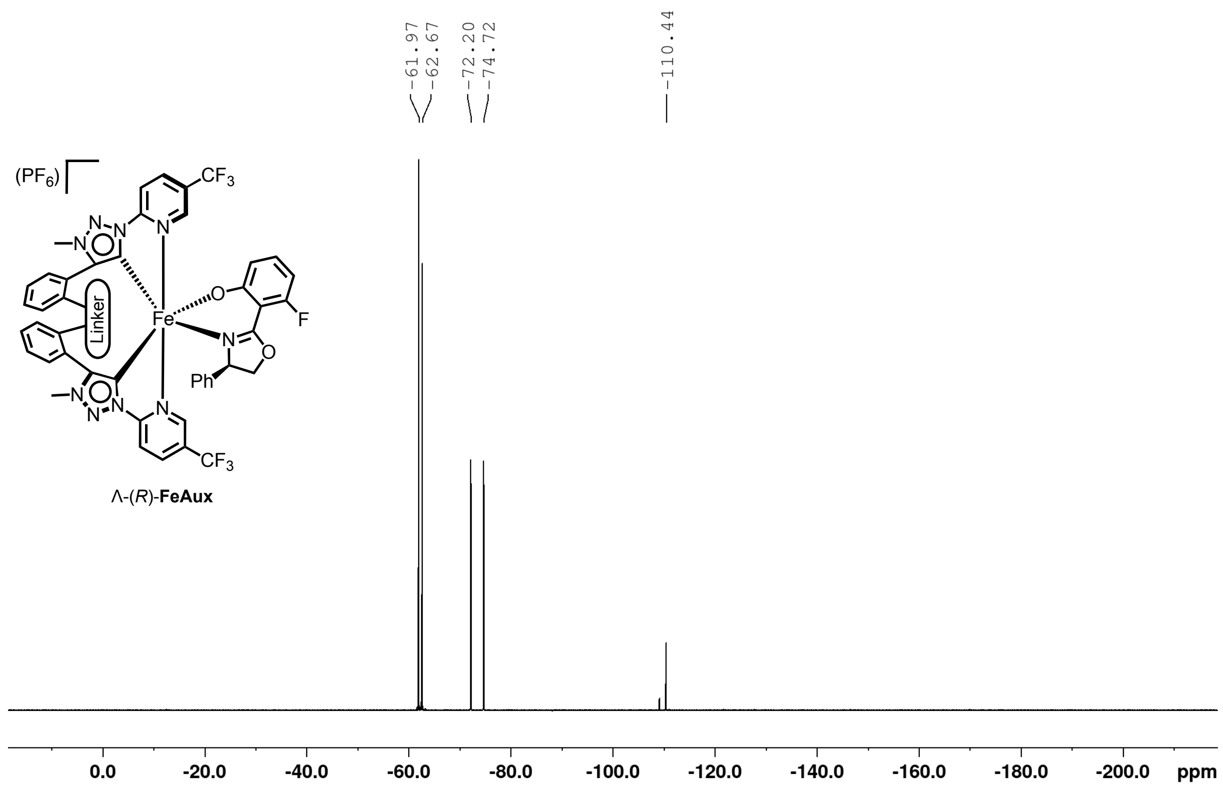

Figure S19: ^{19}F-NMR spectrum of Λ-(*R*)-**FeAux** (282 MHz, CD_2Cl_2, 25 °C).

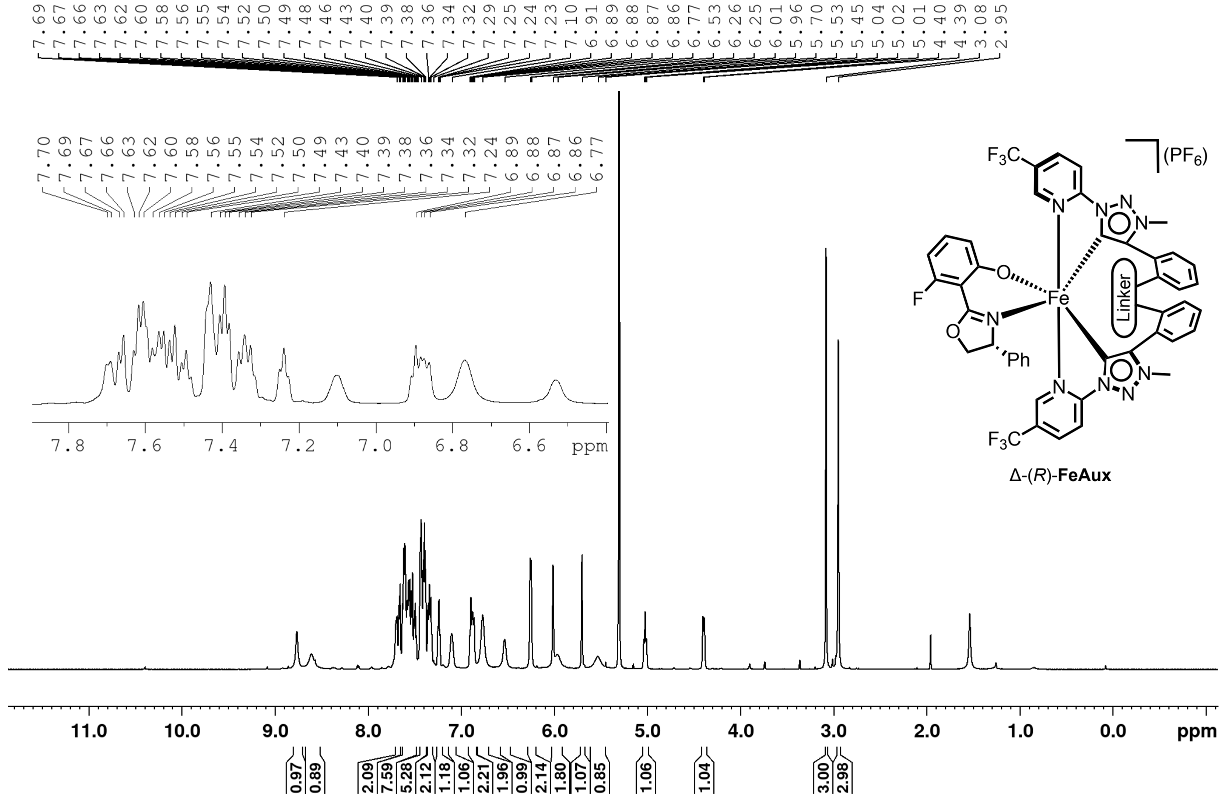

Figure S20: ^{1}H-NMR spectrum of Δ-(*R*)-**FeAux** (600 MHz, CD_2Cl_2, 25 °C).

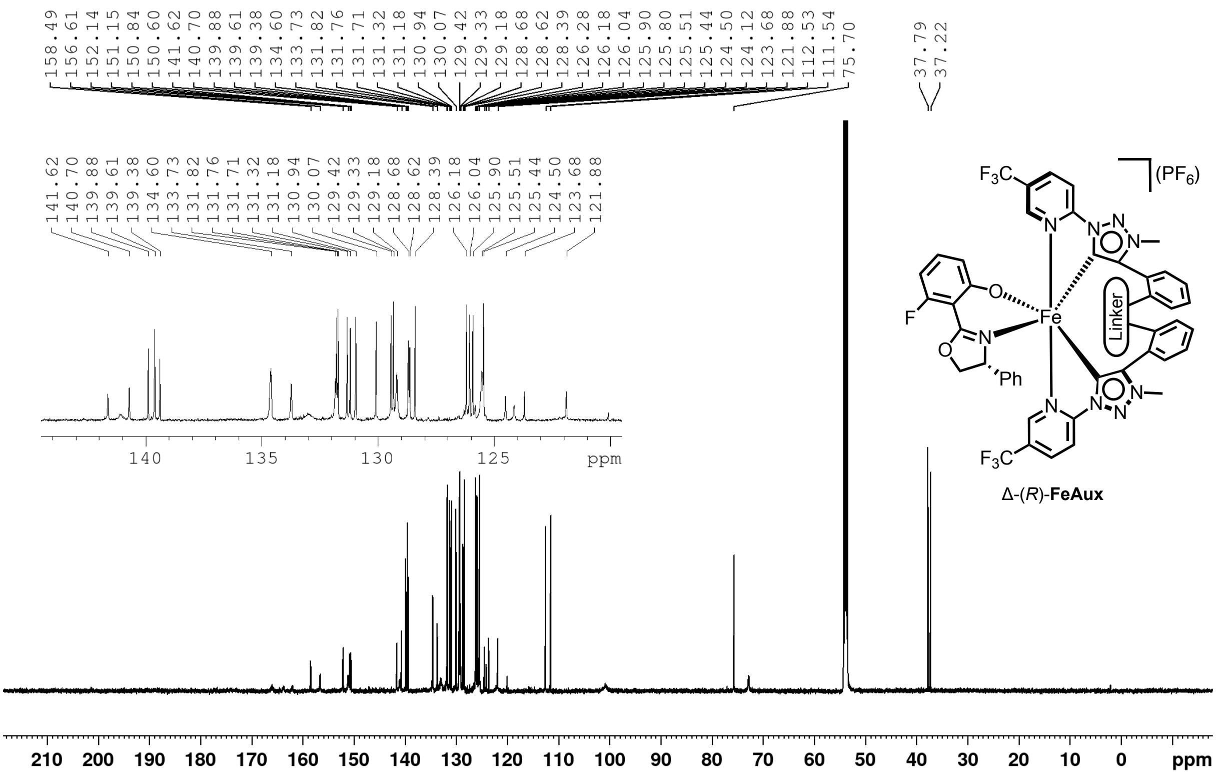

Figure S21: ^{13}C-NMR spectrum of Δ-(*R*)-**FeAux** (151 MHz, CD_2Cl_2, 25 °C).

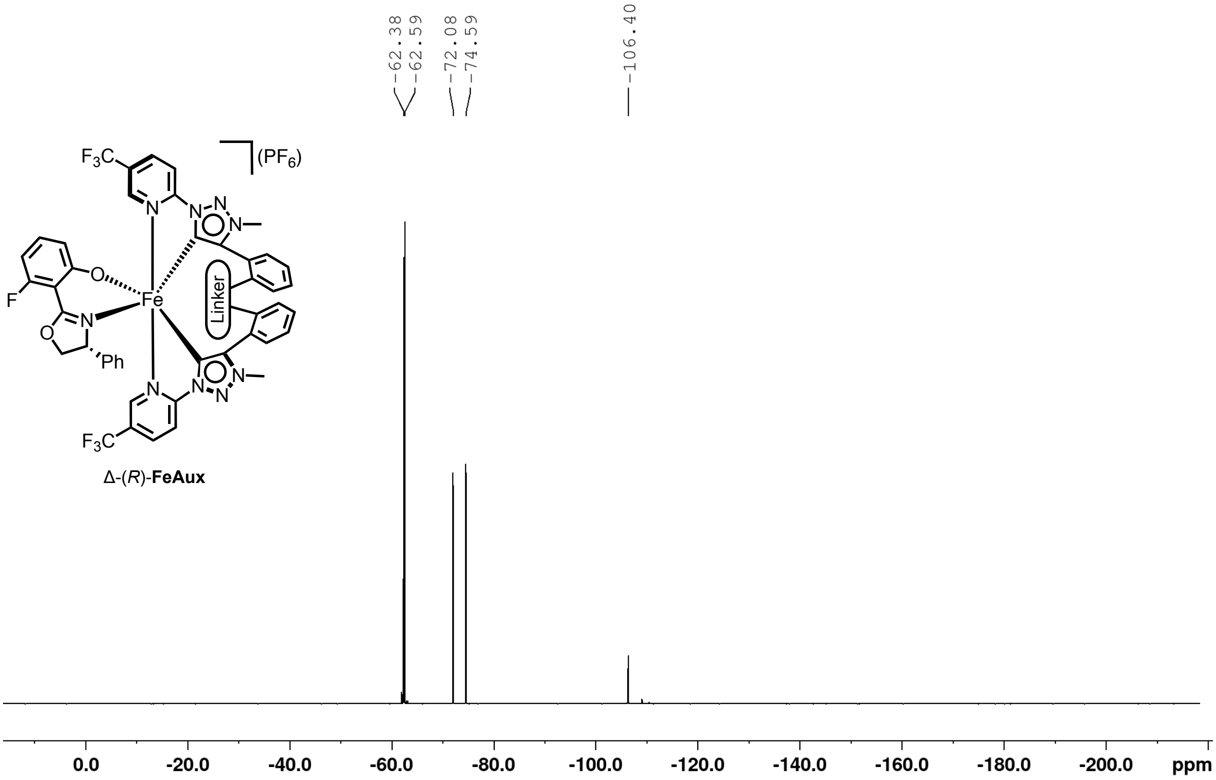

Figure S22: ^{19}F-NMR spectrum of Δ-(*R*)-**FeAux** (282 MHz, CD_2Cl_2, 25 °C).

10. Chiral HPLC Traces

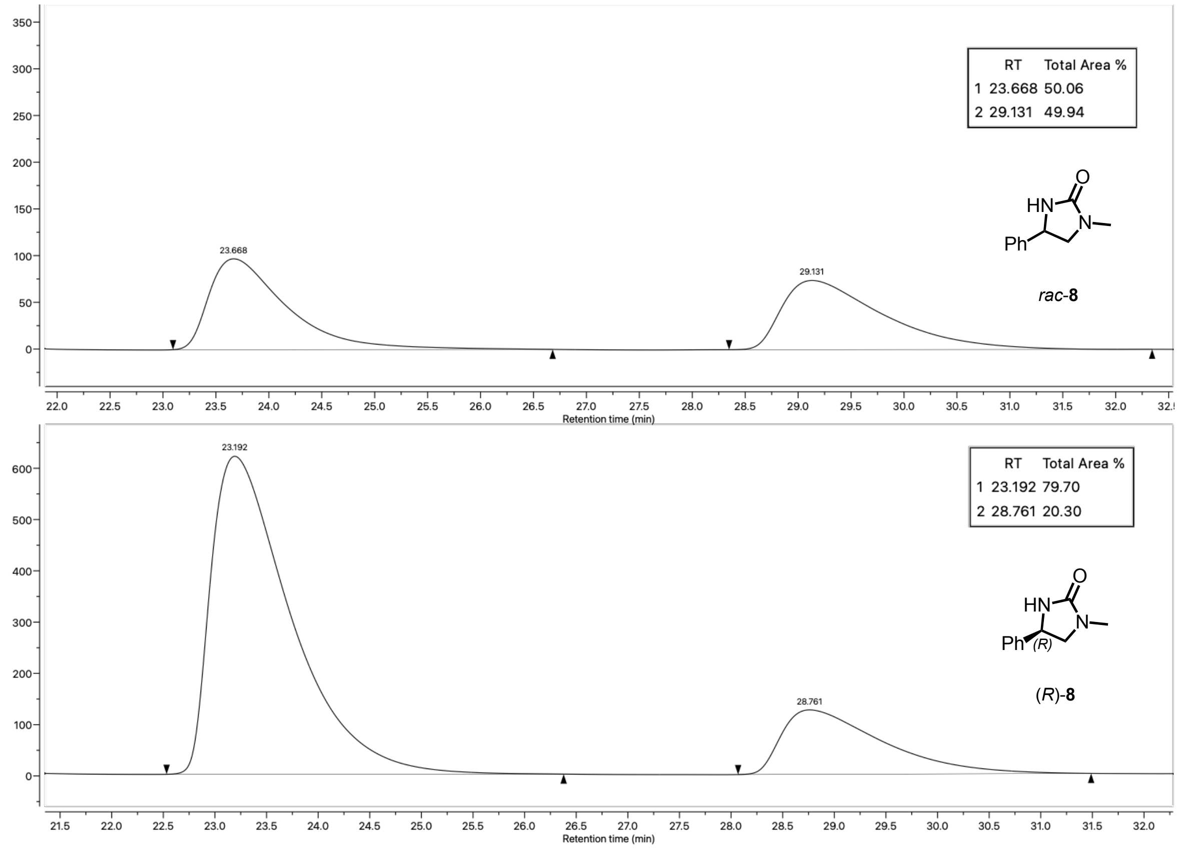

Figure S23: HPLC chromatogram of (*R*)-**8** with 59% ee.

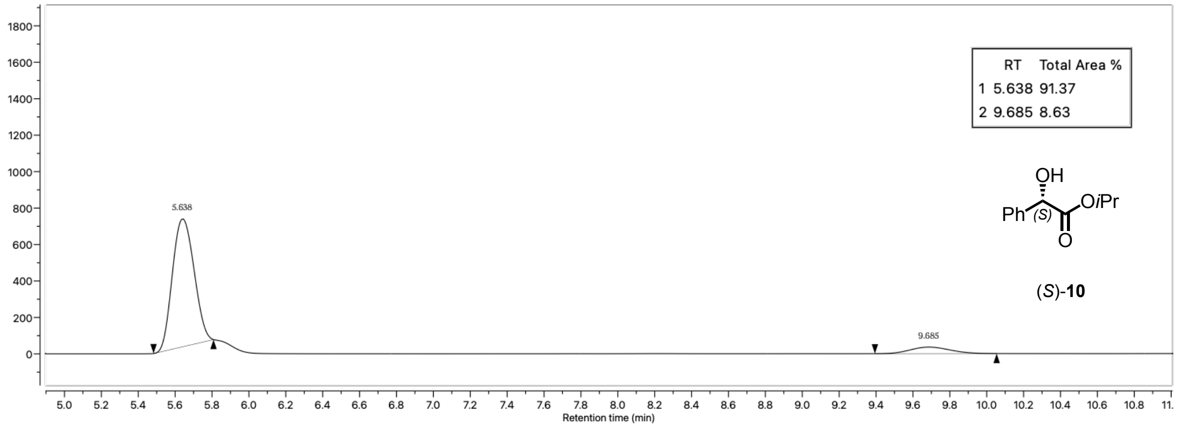

Figure S24: HPLC chromatogram of (*S*)-**10** with 83% ee. The peaks were assigned based on the retention times reported in the literature.[8]

11. CD-Spectra

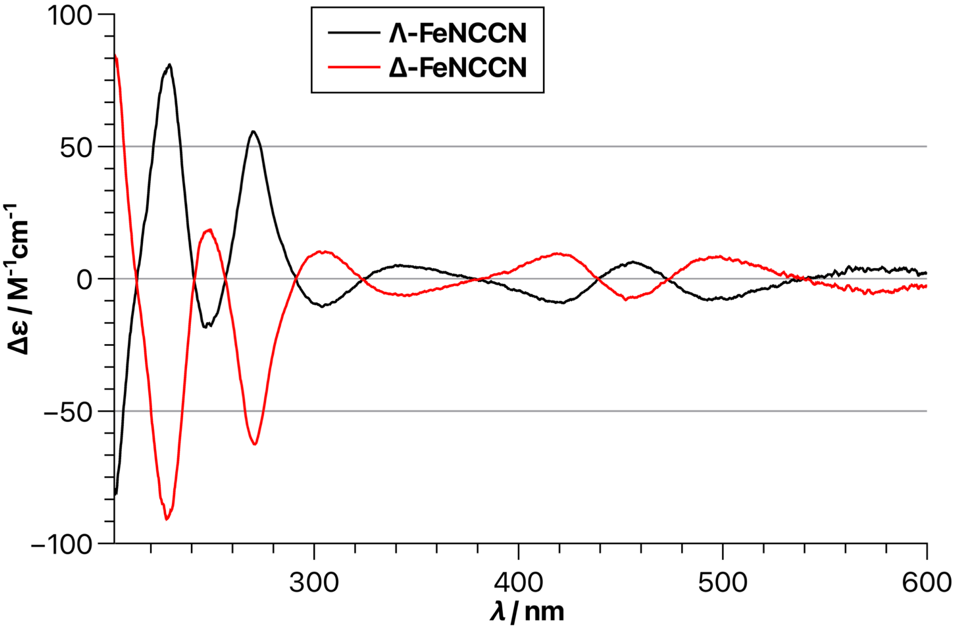

Figure S25: CD-spectra of Λ-**FeNCCN** and Δ-**FeNCCN** in MeCN (0.25 mM).

12. Single Crystal X-Ray Diffraction

***rac*-FeNCCN**

A suitable crystal of $C_{46}H_{34}F_6FeN_{10}(PF_6)_2 \cdot C_2H_3N$ was selected under inert oil and mounted using a MiTeGen loop. Intensity data of the crystal were recorded with a D8 Quest diffractometer (Bruker AXS). The instrument was operated with Mo-Kα radiation (0.71073 Å, microfocus source) and equipped with a PHOTON III C14 detector. Evaluation, integration and reduction of the diffraction data was carried out using the Bruker APEX 5 software suite.[11] Multi-scan and numerical absorption corrections were applied using the SADABS program.[12,13] The structure was solved using dual-space methods (SHELXT-2018/2) and refined against F^2 (SHELXL-2019/1 using ShelXle interface).[14-16] All non-hydrogen atoms were refined with anisotropic displacement parameters. The hydrogen atoms were refined using the "riding model" approach with isotropic displacement parameters 1.2 times (1.5 times for terminal methyl groups) of that of the preceding carbon atom. CCDC 2497296 contains the supplementary crystallographic data for this paper. These data can be obtained free of charge from The Cambridge Crystallographic Data Centre via www.ccdc.cam.ac.uk/structures.

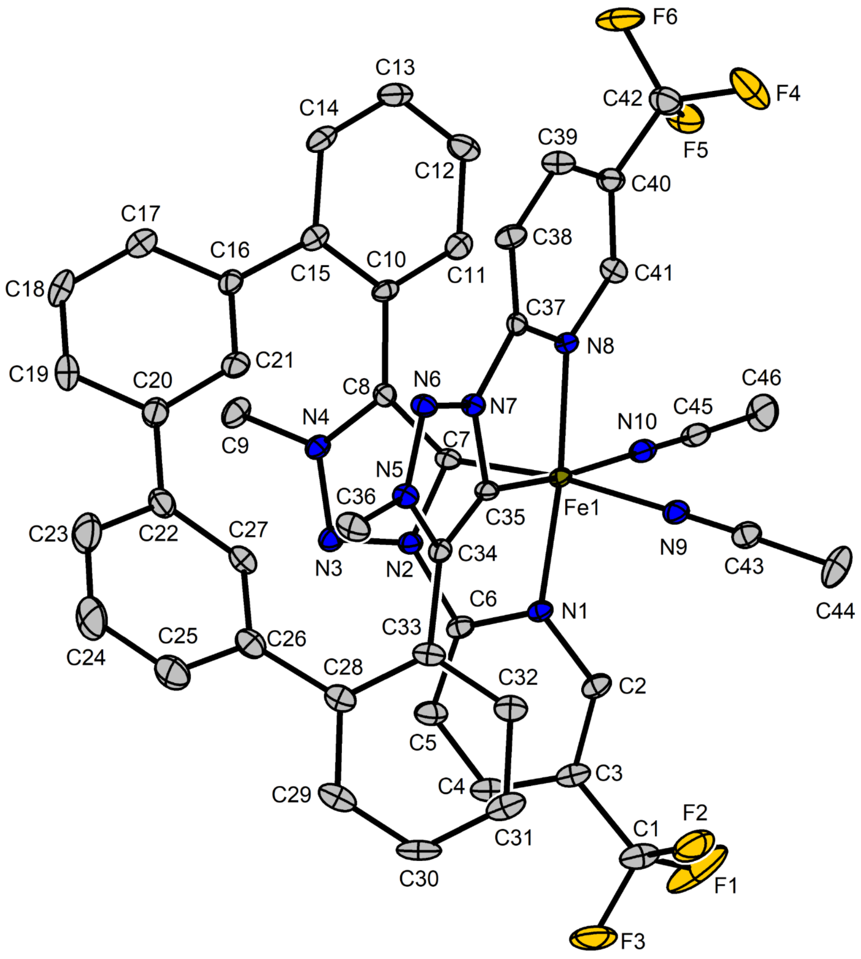

Figure S26: Crystal structure of the *rac*-**FeNCCN** complex. The $[PF_6]^-$anions, hydrogen atoms, and solvent molecules were omitted for clarity. The displacement ellipsoids are shown at a probability level of 50% at 100 K.

Table S1: Selected crystallographic data and details of the structure determination for $C_{46}H_{34}F_6FeN_{10}(PF_6)_2 \cdot C_2H_3N$

Identification code	LHM096
Empirical formula	$C_{48}H_{37}F_{18}FeN_{11}P_2$
Molar mass / g·mol^{-1}	1227.67
Space group (No.)	$P\bar{1}$ (2)
a / Å	12.1386(6)
b / Å	13.8831(6)
c / Å	17.8749(9)
α / °	69.5390(10)
β / °	72.493(2)
γ / °	65.1910(10)
V / Å^3	2519.4(2)
Z	2
$\rho_{calc.}$ / g·cm^{-3}	1.618
μ / mm^{-1}	0.479
Color	dark red
Crystal habitus	plate
Crystal size / mm^3	0.110 x 0.049 x 0.026
T / K	100
λ / Å	0.71073 (Mo-K$_\alpha$)
ϑ range / °	1.879 to 28.304
Range of Miller indices	$-16 \le h \le 16$
	$-18 \le k \le 17$
	$-23 \le l \le 23$
Absorption correction	multi-scan and numerical
T_{min}, T_{max}	0.9506, 0.9912
R_{int}, R_σ	0.0681, 0.0550
Completeness of the data set	0.999
No. of measured reflections	64353
No. of independent reflections	12522
No. of parameters	726
No. of restraints	0
S (all data)	1.105
$R(F)$ ($I \ge 2\sigma(I)$, all data)	0.0600, 0.0800
$wR(F^2)$ ($I \ge 2\sigma(I)$, all data)	0.1102, 0.1176
Extinction coefficient	not refined
$\Delta\rho_{max}$, $\Delta\rho_{min}$ / e·Å^{-3}	0.430, –0.538

Λ-(*R*)-**FeAux**

A suitable crystal of $C_{57}H_{39}F_7FeN_9O_2(PF_6) \cdot CH_2Cl_2$ was selected under inert oil and mounted using a MiTeGen loop. Intensity data of the crystal were recorded with a D8 Venture diffractometer (Bruker AXS). The instrument was operated with Mo-Kα radiation (0.71073 Å, microfocus source) and equipped with a PHOTON III C14 detector. Evaluation, integration and reduction of the diffraction data was carried out using the Bruker APEX 5 software suite.[11] Multi-scan and numerical absorption corrections were applied using the SADABS program.[12,13] The structure was solved using dual-space methods (SHELXT-2018/2) and refined against F^2 (SHELXL-2019/1 using ShelXle interface).[14-16] All non-hydrogen atoms were refined with anisotropic displacement parameters. The hydrogen atoms were refined using the "riding model" approach with isotropic displacement parameters 1.2 times (1.5 times for terminal methyl groups) of that of the preceding carbon atom. CCDC 2497297 contains the supplementary crystallographic data for this paper. These data can be obtained free of charge from The Cambridge Crystallographic Data Centre via www.ccdc.cam.ac.uk/structures.

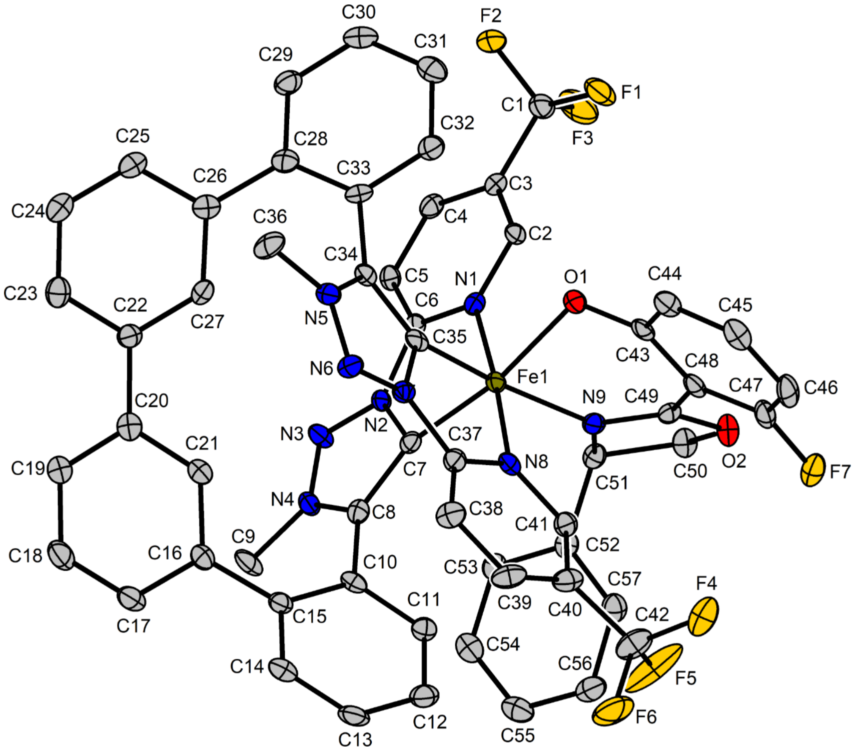

Figure S27: Crystal structure of the Λ-(*R*)-FeAux complex. The $[PF_6]^-$anions, hydrogen atoms, and solvent molecules were omitted for clarity. The displacement ellipsoids are shown at a probability level of 50% at 100 K.

Table S2: Selected crystallographic data and details of the structure determination for $C_{57}H_{39}F_7FeN_9O_2(PF_6) \cdot CH_2Cl_2$

Identification code	LHM181F1
Empirical formula	$C_{58}H_{41}Cl_2F_{13}FeN_9O_2P$
Molar mass / g·mol^{-1}	1300.72
Space group (No.)	$P2_1$ (4)
a / Å	12.0437(3)
b / Å	19.9432(6)
c / Å	12.6380(4)
β / °	115.7200(10)
V / Å^3	2734.78(14)
Z	2
$\rho_{calc.}$ / g·cm^{-3}	1.580
μ / mm^{-1}	0.502
Color	green
Crystal habitus	plate
Crystal size / mm^3	0.105 x 0.076 x 0.019
T / K	100
λ / Å	0.71073 (Mo-K$_\alpha$)
ϑ range / °	1.951 to 25.722
Range of Miller indices	$-14 \le h \le 14$
	$-24 \le k \le 24$
	$-15 \le l \le 15$
Absorption correction	multi-scan and numerical
T_{min}, T_{max}	0.9478, 0.9930
R_{int}, R_σ	0.0737, 0.0436
Completeness of the data set	1.000
No. of measured reflections	80868
No. of independent reflections	10403
No. of parameters	777
No. of restraints	1
S (all data)	1.035
$R(F)$ ($I \ge 2\sigma(I)$, all data)	0.0356, 0.0441
$wR(F^2)$ ($I \ge 2\sigma(I)$, all data)	0.0747, 0.0782
Extinction coefficient	not refined
Flack parameter x	–0.006(7)
$\Delta\rho_{max}$, $\Delta\rho_{min}$ / e·Å^{-3}	0.654, –0.418

Δ-(*R*)-FeAux

A suitable crystal of $C_{57}H_{39}F_7FeN_9O_2(PF_6) \cdot 2\ CH_2Cl_2$ was selected under inert oil and mounted using a MiTeGen loop. Intensity data of the crystal were recorded with a D8 Venture diffractometer (Bruker AXS). The instrument was operated with Mo-Kα radiation (0.71073 Å, microfocus source) and equipped with a PHOTON III C14 detector. Evaluation, integration and reduction of the diffraction data was carried out using the Bruker APEX 5 software suite.[11] Multi-scan and numerical absorption corrections were applied using the SADABS program.[12,13] The structure was solved using dual-space methods (SHELXT-2018/2) and refined against F^2 (SHELXL-2019/1 using ShelXle interface).[14-16] All non-hydrogen atoms were refined with anisotropic displacement parameters. The hydrogen atoms were refined using the "riding model" approach with isotropic displacement parameters 1.2 times (1.5 times for terminal methyl groups) of that of the preceding carbon atom. CCDC 2497298 contains the supplementary crystallographic data for this paper. These data can be obtained free of charge from The Cambridge Crystallographic Data Centre via www.ccdc.cam.ac.uk/structures.

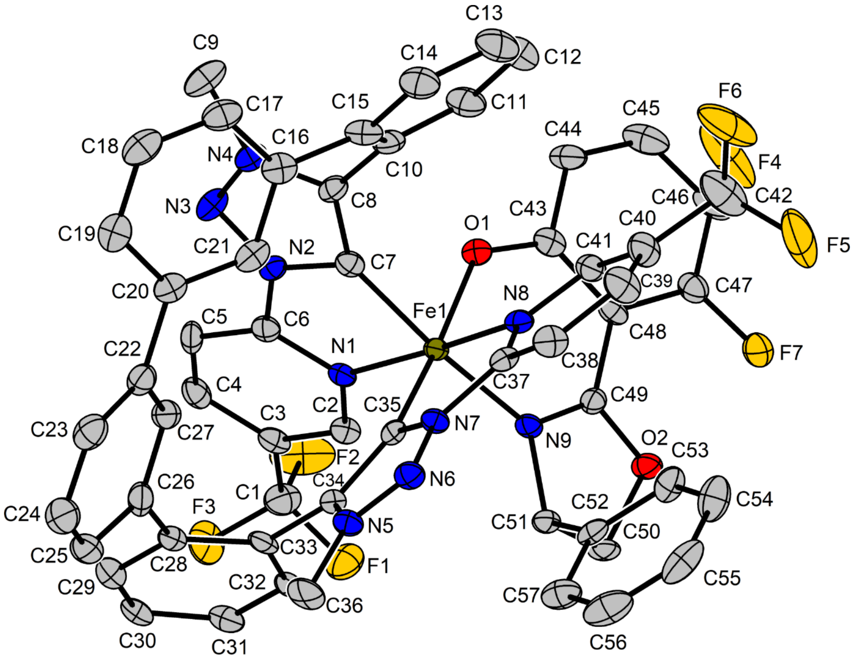

Figure S28: Crystal structure of the Δ-(*R*)-FeAux complex. The $[PF_6]^-$anions, hydrogen atoms, and solvent molecules were omitted for clarity. The displacement ellipsoids are shown at a probability level of 50% at 100 K.

Table S3: Selected crystallographic data and details of the structure determination for $C_{57}H_{39}F_7FeN_9O_2(PF_6) \cdot 2\ CH_2Cl_2$

Identification code	LHM181F2
Empirical formula	$C_{59}H_{43}Cl_4F_{13}FeN_9O_2P$
Molar mass / g·mol^{-1}	1385.64
Space group (No.)	$P2_12_12_1$ (19)
a / Å	13.1706(8)
b / Å	14.2587(9)
c / Å	31.165(2)
V / Å^3	5852.6(6)
Z	4
$\rho_{calc.}$ / g·cm^{-3}	1.573
μ / mm^{-1}	0.562
Color	dark yellow
Crystal habitus	plate
Crystal size / mm^3	0.108 x 0.078 x 0.033
T / K	100
λ / Å	0.71073 (Mo-K$_\alpha$)
ϑ range / °	2.025 to 25.779
Range of Miller indices	$-15 \le h \le 16$
	$-17 \le k \le 17$
	$-38 \le l \le 36$
Absorption correction	multi-scan and numerical
T_{min}, T_{max}	0.9303, 1.0000
R_{int}, R_σ	0.0619, 0.0404
Completeness of the data set	0.999
No. of measured reflections	78698
No. of independent reflections	11181
No. of parameters	805
No. of restraints	0
S (all data)	1.077
$R(F)$ ($I \ge 2\sigma(I)$, all data)	0.0450, 0.0533
$wR(F^2)$ ($I \ge 2\sigma(I)$, all data)	0.1102, 0.1143
Extinction coefficient	0.0016(3)
Flack parameter *x*	0.004(6)
$\Delta\rho_{max}$, $\Delta\rho_{min}$ / e·Å^{-3}	0.454, –0.749

13. References

[1] S. Roy, H. Khatua, S. K. Das, B. Chattopadhyay, *Angew. Chem., Int. Ed*. **2019**, *58*, 11439.

[2] D. Franco, M. Gómez, F. Jiménez, G. Muller, M. Rocamora, M. A. Maestro, J. Mahía, *Organometallics* **2004**, *23*, 3197.

[3] T. Cui, C.-X. Ye, J. Thelemann, D. Jenisch, E. Meggers, *Chin. J. Chem.* **2023**, *41*, 2065.

[4] A. Bolje, D. Urankar, J. Košmrlj, *Eur. J. Org. Chem*. **2014**, *2014*, 8167.

[5] R. Nandi, S. Niyogi, S. Kundu, A. Mondal, N. K. Roy, A. Bisai, *Org. Chem. Front*. **2025**, *12*, 928.

[6] B. Buchberger, N. Demirel, X. Xie, S. I. Ivlev, E. Meggers, *Chem. Commun*. **2025**, *61*, 1894.

[7] N. Demirel, M. Dawor, G. Nadler, S. I. Ivlev, E. Meggers, *Chem. Sci.* **2024**, *15*, 15625.

[8] Y. Hong, L. Jarrige, K. Harms, E. Meggers, *J. Am. Chem. Soc*. **2019**, *141*, 4569.

[9] N. Demirel, P. Moths, X. Xie, S. I. Ivlev, E. Meggers, *Chemistry* **2025**, *31*, e202403792.

[10] Z. Zhou, Y. Tan, T. Yamahira, S. Ivlev, X. Xie, R. Riedel, M. Hemming, M. Kimura and E. Meggers, *Chem*, **2020**, *6*, 2024.

[11] *APEX5*, Bruker AXS Inc., Madison, Wisconsin, USA, **2023**.

[12] *SADABS*, Bruker AXS Inc., Madison, Wisconsin, USA, **2016**.

[13] L. Krause, R. Herbst-Irmer, G. M. Sheldrick, D. Stalke, *J. Appl. Crystallogr*. **2015**, *48*, 3.

[14] G. M. Sheldrick, *Acta Crystallogr., Sect. A: Found. Adv*. **2015**, *71*, 3.

[15] G. M. Sheldrick, *Acta Crystallogr*., *Sect. C: Struct. Chem*. **2015**, *71*, 3.

[16] C. B. Hübschle, G. M. Sheldrick, B. Dittrich, *J. Appl. Crystallogr*. **2011**, *44*, 1281.
